# LIMP-2 enhances cancer stem-like cell properties by promoting autophagy-induced GSK3β degradation in head and neck squamous cell carcinoma

**DOI:** 10.1038/s41368-023-00229-0

**Published:** 2023-06-08

**Authors:** Yuantong Liu, Shujin Li, Shuo Wang, Qichao Yang, Zhizhong Wu, Mengjie Zhang, Lei Chen, Zhijun Sun

**Affiliations:** 1 The State Key Laboratory Breeding Base of Basic Science of Stomatology (Hubei-MOST) & Key Laboratory for Oral Biomedicine Ministry of Education, School and Hospital of Stomatology, Wuhan University, Wuhan, China; 2 Department of Oral Maxillofacial-Head Neck Oncology, School and Hospital of Stomatology, Wuhan University, Wuhan, China

**Keywords:** Oral cancer, Cancer stem cells, Autophagy, Mechanisms of disease

## Abstract

Cancer stem cell-like cells (CSCs) play an integral role in the heterogeneity, metastasis, and treatment resistance of head and neck squamous cell carcinoma (HNSCC) due to their high tumor initiation capacity and plasticity. Here, we identified a candidate gene named LIMP-2 as a novel therapeutic target regulating HNSCC progression and CSC properties. The high expression of LIMP-2 in HNSCC patients suggested a poor prognosis and potential immunotherapy resistance. Functionally, LIMP-2 can facilitate autolysosome formation to promote autophagic flux. LIMP-2 knockdown inhibits autophagic flux and reduces the tumorigenic ability of HNSCC. Further mechanistic studies suggest that enhanced autophagy helps HNSCC maintain stemness and promotes degradation of GSK3β, which in turn facilitates nuclear translocation of β-catenin and transcription of downstream target genes. In conclusion, this study reveals LIMP-2 as a novel prospective therapeutic target for HNSCC and provides evidence for a link between autophagy, CSC, and immunotherapy resistance.

## Introduction

Head and neck squamous cell carcinoma (HNSCC) is characterized by a high degree of heterogeneity and is one of the most common malignancies.^1–4^ Cancer stem-like cells (CSCs) with self-renewal properties and clonal cancer initiation potential play an integral role in the heterogeneity, metastasis, and treatment resistance of HNSCC.^5–7^ Although a large number of studies have explored the characteristics of CSCs in HNSCC, little is known about therapeutic targets to modulate CSC properties and contribute to HNSCC progression. This contradiction highlights an urgent need for elucidating the pivotal pathways promoting CSC properties and therapeutic resistance in HNSCC.

Mounting evidence suggests that autophagy is an intrinsic feature for maintaining stemness and is closely associated with the aggressiveness and chemoresistance of CSCs in various cancers.^8,9^ Autophagy is a lysosome-mediated degradation system used to recycle cellular contents and damaged organelles and thus maintain normal cellular function.^10^ Due to its double-edged property in cancer, autophagy is still a challenge to investigate in depth its role in carcinogenesis and evolution.^10–12^ On the one hand, autophagy serves as a survival mechanism that inhibits malignant transformation by degrading damaged proteins and maintaining genomic stability. On the other hand, autophagy facilitates cancer cells to maintain cellular homeostasis under adverse conditions such as malnutrition and hypoxia which favors cancer progression. In addition to maintaining cellular homeostasis, autophagy can influence malignant behaviors, such as the epithelial-mesenchymal transition (EMT) process and CSC properties, which in turn promote cancer development and metastasis.^9^ Autophagy is significantly upregulated in CSCs and represents a promising target for counteracting CSC aggressiveness in HNSCC.^13^

Lysosomal integral membrane protein type 2 (LIMP-2, also named SCARB2) belongs to the CD36 family and is essential for the proper function and maintenance of both lysosomes and endosomes.^14^ As a phospholipid receptor, LIMP-2 regulates lysosomal cholesterol export.^15,16^ In addition, LIMP-2 serves as a specific receptor for glucocerebrosidase (GCase) and has been shown to be involved in the development of Gaucher’s disease.^17^ Recent studies have shown that higher expression of LIMP-2 in glioma cells, which is related to enterovirus A71-induced oncolysis.^18,19^ However, the function of LIMP-2 in cancer, especially in HNSCC, has not been well studied.

In this study, we identified LIMP-2 as a potential regulator of HNSCC progression based on a bioinformatic screen of genes involved in the autophagy‒lysosome pathway, which was further validated by clinical samples and in vitro and in vivo results. Mechanistically, LIMP-2 regulated HNSCC progression and stemness by promoting the formation of autolysosomes and inducing Wnt/β-catenin pathway activation. Further results suggest that LIMP-2 promotes Wnt/β-catenin signaling activation by regulating the autophagy-induced GSK3β degradation pathway. Our data comprehensively described the mechanism of cancer progression via the LIMP-2 signaling axis, revealing new insights into the link between autophagy, CSC, and immunotherapy resistance.

## Results

### Bioinformatics screening based on the autophagy‒lysosome pathway reveals LIMP-2 as a potential regulator of HNSCC progression

**Fig. 1:**
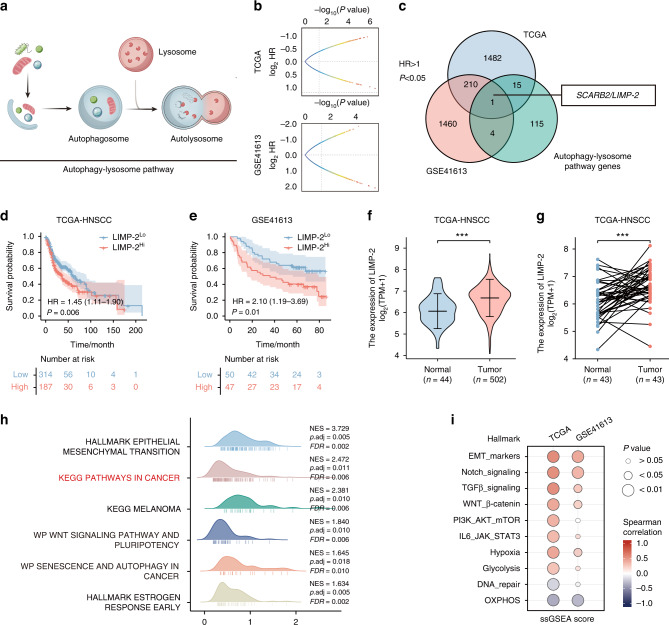
Bioinformatics results identified LIMP-2 as a potential regulator of HNSCC progression. **a** Genes involved in autophagy‒lysosome biological processes (*n* = 135) were identified. The autophagy‒lysosome pathway plays a significant role in the development of cancer. **b** Cox regression analysis was used to screen the TCGA-HNSCC and GSE41613 datasets for genes significantly correlated with worse overall survival (*P* < 0.05, HR > 1, median histoscore as cutoff value). **c** LIMP-2 is shown in the set of genes associated with the autophagy‒lysosome pathway and in two independent sets of genes associated with poor prognosis in HNSCC. Kaplan‒Meier survival analysis suggests that high expression of LIMP-2 is associated with poor prognosis at the best cutoff value in the (**d**) TCGA-HNSCC dataset and (**e**) GSE41613 dataset. LIMP-2 mRNA expression was upregulated in the HNSCC group compared with its (**f**) normal counterpart and (**g**) paired cancer-adjacent tissues in the TCGA-HNSCC dataset. **h** GSEA analysis of LIMP-2 based on the TCGA-HNSCC dataset. **i** Spearman’s correlation of LIMP-2 with cancer hallmark gene sets was analyzed in TCGA-HNSCC and GSE41613 databases using the ssGSEA method. ^*^P 0.05, ^**^P < 0.01, and ^***^P < 0.001

The autophagy‒lysosome pathway is involved in the regulation of cellular homeostasis and malignant phenotype of cancer. We identified 135 genes from a comprehensive autophagy‒lysosome gene signature based on data from existing studies (Fig. 1a).^20^ Subsequently, we screened the TCGA-HNSCC and GSE41613 databases for genes involved in unfavorable overall survival (OS) by Cox regression analysis (*p* < 0.05, HR > 1, median histoscore as cutoff value) (Fig. 1b). We found that among the genes related to the autophagy–lysosome pathway, only LIMP-2 overexpression was associated with a poor prognosis for HNSCC patients in both independent datasets (Fig. 1c–e, S1c).

Then, we found higher LIMP-2 levels in HNSCC tissues compared to its normal counterpart and paired cancer-adjacent tissues in the TCGA-HNSCC dataset (Fig. 1f, g). Additionally, we confirmed LIMP-2 expression in cancer and normal samples across cancers using TCGA and GTEx data. Significant upregulation was observed in 23 cancers including HNSCC (Fig. S1d). Through univariate and multivariate Cox regression analyses, LIMP-2 expression was identified as an independent prognostic factor for OS in HNSCC patients (Table 1).

**Table 1:** Univariate and multivariate Cox regression analyses of overall survival in TCGA-HNSCC dataset

Characteristics	Univariate analysis		Multivariate analysis	
	*P*	HR (95% CI)	*P*	HR (95% CI)
Age (Old vs. Young)	0.102	1.252 (0.956–1.639)		
Gender (Female vs. Male)	0.066	1.309 (0.983–1.743)	0.046^*^	1.342 (1.005–1.791)
Histologic grade (III + IV vs. I + II)	0.692	0.939 (0.688–1.282)		
T stage (T3 + T4 vs. T1 + T2)	0.137	1.245 (0.932–1.661)		
N stage (N1 + N2 + N3 vs. N0)	0.09	1.263 (0.964–1.653)	0.072	1.281 (0.978–1.678)
Clinical stage (III + IV vs. I + II)	0.238	1.217 (0.878–1.688)		
Smoker	0.618	1.089 (0.778–1.525)		
Alcohol history	0.733	0.952 (0.716–1.265)		
LIMP-2 expression (Continuous)	0.024	1.264 (1.032–1.548)	0.02^*^	1.278 (1.040–1.570)

*HR* hazard ratio, *CI* confidence interval

^*^P < 0.05

To elucidate the possible biological role of LIMP-2 in HNSCC, we performed GSEA to identify the gene sets enriched in different LIMP-2 expression subgroups. The results of enrichment analysis indicated that LIMP-2 may be closely associated with cancer progression as well as multiple oncogenic pathways (Fig. 1h). In addition, we evaluated the correlation between LIMP-2 and cancer hallmark gene sets based on the TCGA-HNSCC and GSE41613 datasets using ssGSEA. The results revealed that LIMP-2 was positively correlated with cancer hallmarks such as the EMT program, TGFβ signaling, Wnt signaling, and hypoxia, but negatively correlated with hallmarks such as oxidative phosphorylation (OXPHOS) (Fig. 1i). Overall, the bioinformatics results suggested that LIMP-2, an autophagy‒lysosome candidate gene, may be associated with HNSCC progression.

### LIMP-2 overexpression is associated with the progression and poor prognosis of HNSCC

**Fig. 2:**
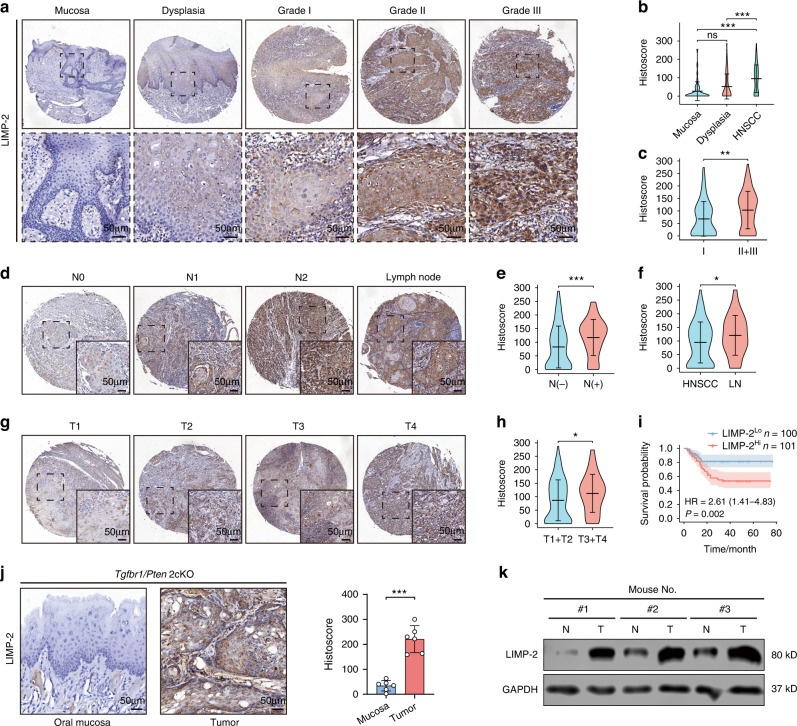
LIMP-2 is upregulated in HNSCC tissues and predicts a poor prognosis. **a** Representative IHC staining results of LIMP-2 in normal mucosa, dysplasia, and HNSCC samples with different pathological grades (scale bar, 50 μm). **b** Quantification of LIMP-2 expression in HNSCC (*n* = 210) compared with dysplasia (*n* = 69) and oral mucosa (*n* = 42) by IHC analysis. **c** Quantification of the LIMP-2 histoscore between pathological grade I (*n* = 53) and pathological grade II + III (*n* = 157). **d** Representative IHC results of LIMP-2 in tumor samples of different N stage classifications (scale bar, 50 μm). **e** Quantification of LIMP-2 expression between N0 (*n* = 138) and N1 + N2 (*n* = 72). **f** Quantification of LIMP-2 expression in primary cancer samples (*n* = 210) and metastatic lymph nodes (*n* = 54). **g** Representative IHC results of LIMP-2 in tumor samples of different T stage classifications (scale bar, 50 μm). **h** Quantification of LIMP-2 expression between T1 + T2 (*n* = 145) and T3 + T4 (*n* = 65). **i** Kaplan‒Meier survival analysis suggested that high expression of LIMP-2 was associated with unfavourable prognosis (median histoscore as cutoff value). **j** Representative IHC results of LIMP-2 in normal oral mucosal tissues compared with *Tgfbr1/Pten* 2cKO HNSCC mouse tissues (scale bar, 50 μm). **k** LIMP-2 expression in HNSCC mouse samples was significantly higher than that in normal oral mucosa detected by western blot. The bar values represent the SD. ^*^P < 0.05, ^**^P < 0.01 and ^***^P < 0.001

Based on bioinformatics screening data, we decided to examine the role of LIMP-2 in cancer, especially in HNSCC, as the gene may be responsible for the progression and poor prognosis. To validate the results of the bioinformatics analysis, we investigated LIMP-2 expression in the HNSCC tissue microarray (TMA) by immunohistochemistry (IHC) staining. Consistent with public data, LIMP-2 expression was significantly higher in HNSCC tissue than in dysplasia and normal mucosa (Fig. 2a, b). Further analysis showed that LIMP-2 expression was positively correlated with poor pathologic grade, lymph node metastasis, and large cancer size (Fig. 2a–h, S2a). In addition, cancer tissue in metastatic lymph nodes has higher IHC histoscore compared to primary cancer tissue (Fig. 2f). Past studies have suggested that estrogen may regulate LIMP-2 expression in hepatocytes at the RNA splicing and posttranslational levels.^21^ Interestingly, in our patient cohort, women tended to have higher expression of LIMP-2 than men, which was also demonstrated in the GSE41613 dataset (Fig. S2b, c). However, LIMP-2 expression was not associated with other clinical parameters such as age, smoking, alcohol consumption, or radiotherapy (Table S1, Fig. S2d). Survival analyses suggested high LIMP-2 expression predicted poor clinical outcomes in HNSCC patients (Fig. 2i, S2e). Meanwhile, the expression of LIMP-2 was found to be an independent risk factor affecting the OS of HNSCC patients, analyzing multivariate Cox after modifying for potential confounding factors (Table 2).

**Table 2:** Univariate and multivariate Cox regression analyses of overall survival in our patient cohort

Characteristics	Univariate analysis		Multivariate analysis	
	*P*	HR (95% CI)	*P*	HR (95% CI)
Age (Old vs. Young)	0.026	1.879 (1.079–3.273)	0.034^*^	1.825 (1.047–3.183)
Gender (Female vs. Male)	0.281	1.402 (0.758–2.594)		
Pathological grade (III vs. I + II)	0.993	1.003 (0.502–2.004)		
T stage (T3 + T4 vs. T1 + T2)	0.016	1.982 (1.138–3.451)	0.106	1.597 (0.906–2.815)
N stage (N1 + N2 vs. N0)	0.033	1.817 (1.048–3.151)	0.26	1.387 (0.785–2.453)
LIMP-2 expression (High vs. Low)	0.002	2.610 (1.410–4.830)	0.016^*^	2.184 (1.156–4.126)

*HR* hazard ratio, *CI* confidence interval

^*^p < 0.05

We further examined the expression of LIMP-2 in mouse HNSCC cancer tissues by IHC and western blot assays. Tissue specific *Tgfbr1/Pten* 2cKO mice, a spontaneously developed tumor model, exhibit many of the same biochemical changes as human HNSCC.^22^ The results showed that LIMP-2 expression was obviously elevated in *Tgfbr1/Pten* 2cKO HNSCC tissues compared with normal oral mucosal tissues (Fig. 2j, k). Overall, these results suggest that LIMP-2 expression was significantly elevated in HNSCC and that its levels was an independent risk factor for poor survival in HNSCC patients.

### Knockdown of LIMP-2 blocked autophagic flux by inhibiting the formation of autolysosomes

Three mouse HNSCC cell lines were used to explore the role of LIMP-2 in HNSCC progression. 4MOSC1 and 4MOSC2 cells were derived from 4NQO-induced tongue tumors isolated from female C57BL/6 mice.^23^ Control vectors (Vector) and pcDNA3.1/LIMP-2 plasmid vectors (OE-LIMP-2) were used to establish LIMP-2-overexpressing 4MOSC1 cells, while LIMP-2-targeting shRNA (shLIMP-2) or corresponding controls (shNC) were introduced into 4MOSC2 and SCC7 cells with relatively high endogenous LIMP-2 expression (Fig. S3a, b).

**Fig. 3:**
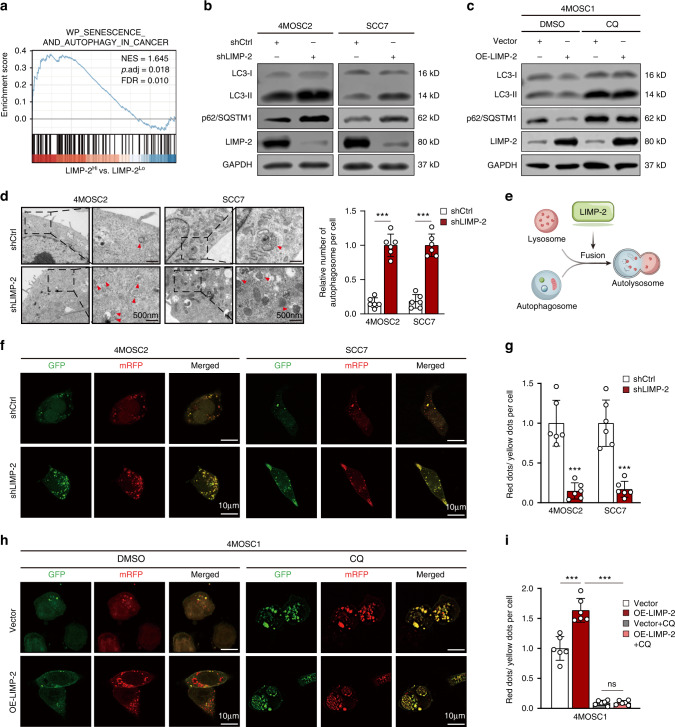
LIMP-2 promotes autophagy in HNSCC by participating in the formation of autolysosomes. **a** GSEA of the TCGA-HNSCC dataset showed that high expression of LIMP-2 was significantly associated with the autophagy pathway in cancers. **b** Western blot analysis showed that LIMP-2 knockdown enhanced the protein levels of LC3-II and p62 compared to those in the negative control groups in 4MOSC2 and SCC7 cells. **c** Western blot analysis of LC3-II, p62, and LIMP-2 in vector and OE-LIMP-2 4MOSC1 cells with or without the autophagy inhibitor chloroquine (CQ). **d** Electron micrographs showed increased accumulation of autophagosomes (red arrow) in 4MOSC2 cells and SCC7 cells after LIMP-2 knockdown (scale bar, 500 nm). **e** LIMP-2 promotes autophagic flux in HNSCC by participating in the formation of autolysosomes but not autophagosomes. **f**, **g** Representative images of mRFP-GFP-LC3 puncta in 4MOSC2 and SCC7 cells with or without LIMP-2 knockdown (scale bars, 10 μm). **h**, **i** Representative images of mRFP-GFP-LC3 puncta in vector and OE-LIMP-2 4MOSC1 cells with or without CQ treatment (scale bars, 10 μm). The ratio of the number of red dots (autolysosomes) to yellow dots (autophagosomes) was evaluated. All results were calculated in at least 3 independent experiments and expressed as mean ± SD. ^*^P < 0.05, ^**^P < 0.01, ^***^P < 0.001, and ns represents no significance

Although lysosomes serve as the ultimate site of autophagy, the lysosomal membrane protein LIMP-2 in the cancer autophagy remains unclear.^24^ GSEA results of the TCGA-HNSCC dataset indicated that LIMP-2 was significantly associated with the autophagy pathway in cancers (Fig. 3a). LIMP-2 was significantly positively correlated with the autophagy indicator LAMP1 in our patient cohort (Fig. S3c). Furthermore, TCGA dataset analysis indicated that LIMP-2 was generally positively correlated with autophagy-related genes in a variety of cancers (Fig. S4).

To investigate the specific mechanism by which LIMP-2 regulates autophagy, we measured the expression of both the autophagic marker LC3B-II and the autophagic substrate p62/SQSTM1. The expression of LC3B-II and p62 was significantly upregulated in LIMP-2-deficient 4MOSC2 cells and was alleviated in LIMP-2-overexpressing 4MOSC1 cells (Figs. 3b, c and S3d, e). However, the expression levels of key molecules involved in autophagosome formation, ATG5 and ATG7, were not remarkably altered in comparison with negative control (NC) groups (Fig. S3f). Using electron micrographs, we observed an increased accumulation of autophagosomes in cancer cells after LIMP-2 knockdown (Fig. 3d). Therefore, we hypothesized that this phenomenon might be due to autophagic flux inhibition rather than the initiation of autophagosome formation (Fig. 3e). To further validate whether autophagic flux was blocked, the mRFP-GFP-LC3 plasmid was transfected to detect autophagic flux (Fig. S3g). The ratio of autolysosomes (red dots) to autophagosomes (yellow dots) was remarkably lower in the LIMP-2 knockdown group than in the shNC group, indicating that autophagic flux was impaired after LIMP-2 knockdown (Fig. 3f, g). In contrast, ectopic LIMP-2 expression in 4MOSC1 cells increased the ratio of autolysosomes to autophagosomes (Fig. 3h, i). Chloroquine (CQ) increased autophagosomes, but not autolysosomes, in control 4MOSC1 cells. CQ also significantly reduced autolysosomes and increased autophagosomes in LIMP-2-overexpressing 4MOSC1 cells, implying that the autophagic flux induced by LIMP-2 overexpression was blocked (Fig. 3h, i). Collectively, these data suggest that LIMP-2 is involved in the formation of autolysosomes in HNSCC.

### Targeting LIMP-2 impairs the self-renewal potential of the CSCs of HNSCC in vitro

**Fig. 4:**
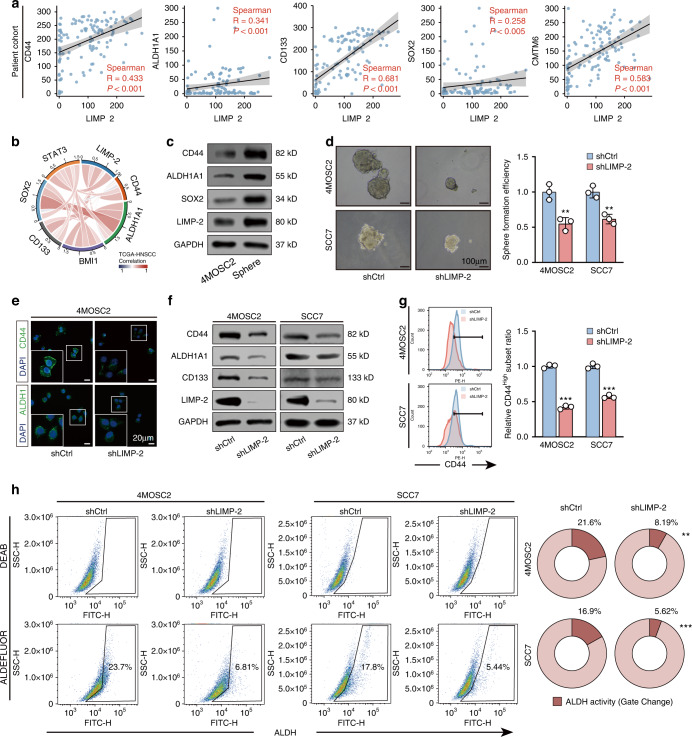
LIMP-2 regulates the cancer stemness of HNSCC cells. **a** Spearman’s correlation between LIMP-2 expression and CD44, ALDH1A1, CD133, SOX2, and CMTM6 in our patient cohort. **b** LIMP-2 was positively correlated with CSC-related markers in the TCGA-HNSCC dataset. **c** LIMP-2 expression was significantly increased in 4MOSC2 sphere cells compared to 4MOSC2 primary adhesion cells. **d** In the sphere formation assay, LIMP-2 knockdown was found to significantly inhibit the self-renewal ability of 4MOSC2 and SCC7 cells in vitro (scale bars, 100 μm). **e** Immunofluorescence images show the expression of CD44 and ALDH1A1 in 4MOSC2 cells with or without LIMP-2 knockdown (scale bar, 20 μm). **f** Western blot assays showed the expression of CSC-related markers in 4MOSC2 and SCC7 cells with or without LIMP-2 knockdown. **g** Flow cytometry analysis was applied to detect CD44^+^ HNSCC cells under LIMP-2 knockdown. **h** ALDH activity in 4MOSC2 and SCC7 cells was analyzed by flow cytometry. ^*^P < 0.05, ^**^P < 0.01 and ^***^P < 0.001

Autophagy induction promotes the acquisition of stemness of non-CSCs and maintains the self-renewal capacity of the CSCs.^8,9^ In view of the close connection between autophagy and CSCs, we then examined the effect of LIMP-2 on HNSCC stemness. The results showed that LIMP-2 was positively correlated with CSC-related markers (CD44, ALDH1A1, CD133, SOX2, and CMTM6) in our patient cohort (Fig. 4a). The close association between LIMP-2 expression and CSC-related markers was also confirmed in the TCGA-HNSCC dataset (Fig. 4b). Subsequently, we found that LIMP-2 was highly expressed in 4MOSC2 tumor sphere cells characterized by strong stemness (Fig. 4c). The in vitro sphere formation assay revealed that LIMP-2 knockdown resulted in the formation of fewer and smaller tumor spheres (Fig. 4d). Similarly, immunofluorescence and western blot analyses revealed that LIMP2 knockdown significantly downregulated the expression of CSC markers (Fig. 4e, f). This finding was confirmed by flow cytometry analysis: the proportion of HNSCC cells with ALDH^+^ and CD44^+^ subpopulations was significantly decreased after LIMP-2 knockdown (Fig. 4g, h). Collectively, these results suggest that LIMP-2 is required to maintain the CSC phenotype in HNSCC.

### LIMP-2 shows a significant effect on HNSCC tumorigenicity

**Fig. 5:**
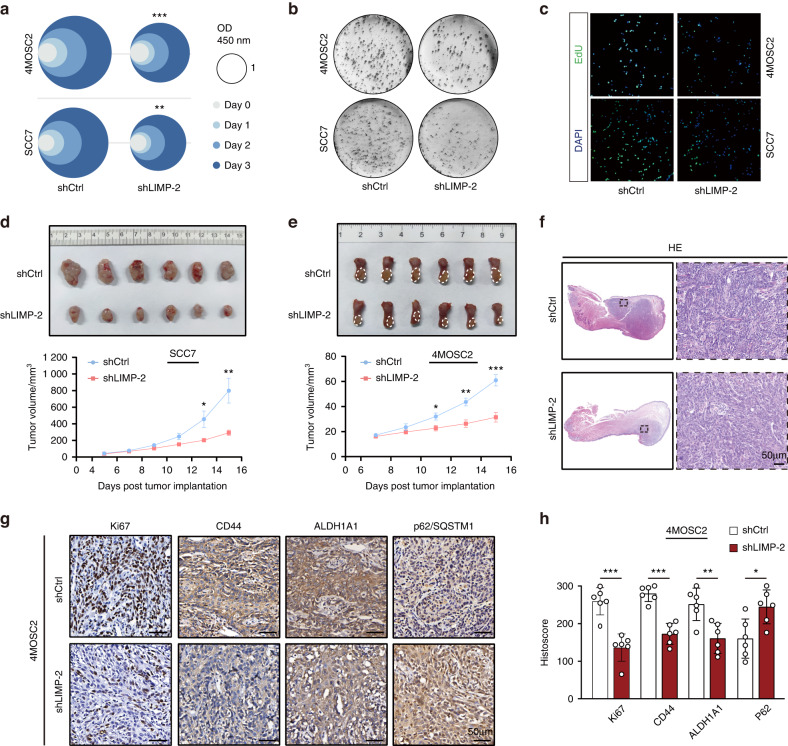
LIMP-2 shows a significant effect on HNSCC tumorigenicity. **a** Relative viability of 4MOSC2 and SCC7 cells. **b** Representative photographs of stained colonies in shLIMP-2 and shCtrl cells. **c** EdU-positive cells were visualized by EdU retention assays. **d**, **e** Tumor growth in the 4MOSC2 orthotopic model and SCC7 subcutaneous allograft model was measured. **f** Representative images of HE staining of LIMP-2 in tumor sections of 4MOSC2 orthotopic HNSCC mouse models (scale bar, 50 μm). **g**, **h** Representative IHC results of Ki67, CD44, ALDH1A1, and p62/SQSTM1 in tumor sections from 4MOSC2 HNSCC mouse models (scale bar, 50 μm). ^*^P < 0.05, ^**^P < 0.01, ^***^P < 0.001, and ns represents no significance

Due to their high self-renewal capacity and plasticity, HNSCC CSCs are prone to metastasis, resulting in treatment resistance.^25^ We found that the viability and clonogenic ability of HNSCC cells in the shLIMP-2 group was significantly reduced compared to those in the shNC group (Figs. 5a, b and S5a). In line with the above results, the number of EdU^+^ 4MOSC2 and SCC7 cells in the LIMP-2-knockdown group was markedly downregulated (Fig. 5c). Moreover, apoptosis was promoted in LIMP-2-knockdown HNSCC cells (Fig. S5b, c). In conclusion, these data support the tumor-promoting role of LIMP-2 in HNSCC cells.

To validate the role of LIMP-2 in vivo, we used 4MOSC2 orthotopic and SCC7 ectopic models for tumorigenesis. The results showed that LIMP-2 knockdown significantly inhibited cancer growth in the 4MOSC2 and SCC7 allograft models (Fig. 5d, e). Consistent with the in vitro findings, reduced Ki67 expression was observed in shLIMP-2 4MOSC2 cell-derived tumor grafts compared to the shNC group, indicating the crucial role of LIMP-2 in the malignant phenotype of HNSCC (Fig. 5f–h). Furthermore, the knockdown of LIMP-2 contributed to reduced expression of CD44 and ALDH1A1, as well as elevated expression of p62, validating the molecular basis identified in vitro (Fig. 5g, h).

### LIMP-2 promotes the stemness of HNSCC in an autophagy-dependent manner

The findings from in vitro and in vivo results, in combination with the analysis of patient specimens, suggested that LIMP-2 could promote the autophagy, stemness, and progression of HNSCC. Recently, evidence has accumulated that targeting autophagy effectively counteracts the aggressiveness of CSCs in HNSCC.^13^ To clarify the role of autophagy in the LIMP-2-induced malignant phenotype of HNSCC, we inhibited autophagy in LIMP-2-overexpressing 4MOSC1 cells with CQ.

Interestingly, the results revealed that CQ suppressed the proliferation and colony formation ability in 4MOSC1 cells transfected with LIMP-2 (Fig. S6a, b). The western blot and immunofluorescence results demonstrated that the overexpression of LIMP-2 increased the expression of CSC-related markers, which were significantly impaired by CQ in 4MOSC1 cells (Fig. S6c, d). Consistently, we confirmed that CQ inhibited the tumor sphere formation ability of LIMP-2 overexpressing 4MOSC1 cells (Fig. S6e). Taken together, these results suggest that autophagy is involved in the LIMP-2-driven malignant phenotype of HNSCC.

### LIMP-2 exerts oncogenic effects by activating the GSK3β/β-catenin pathway

**Fig. 6:**
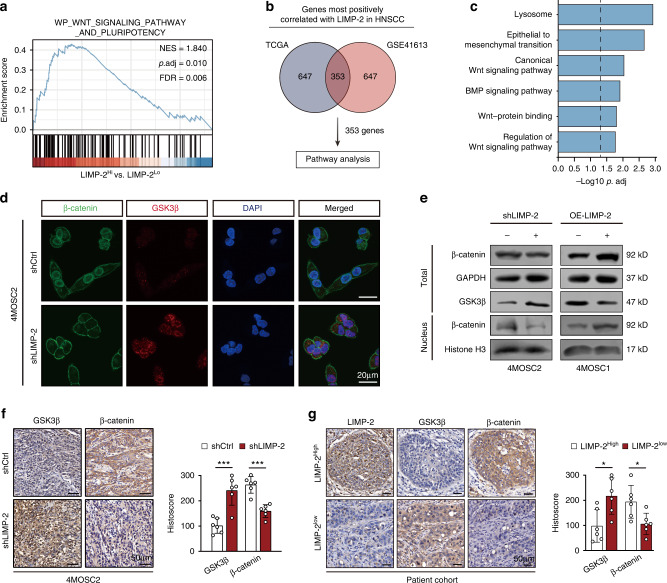
LIMP-2 exerts oncogenic effects by activating the GSK3β/β-catenin pathway. **a** GSEA of the TCGA-HNSCC dataset showed high enrichment of the Wnt signaling pathway and pluripotency in patients with high expression of LIMP-2. **b** Scheme of criteria for identifying genes positively correlated with LIMP-2 in two independent cohorts of HNSCC clinical datasets (TCGA-HNSCC, GSE41613). **c** Gene ontology analyses of LIMP-2-associated genes. **d** Representative immunofluorescence images of β-catenin (green) and GSK3β (red) (Scale bar, 20 μm). **e** Western blot results of the indicated proteins in 4MOSC1 and 4MOSC2 cells. Histone H3 was loaded as a nuclear marker. **f** Representative IHC results of GSK3β and β-catenin in tumor sections of 4MOSC2 orthotopic HNSCC mouse models (scale bar, 50 μm). **g** Representative IHC results of LIMP-2, GSK3β, and β-catenin in HNSCC samples (scale bar, 50 μm). ^*^P < 0.05, ^**^P < 0.01, ^***^P < 0.001, and ns represents no significance

Next, we attempted to identify the downstream factors responsible for the maintenance of HNSCC stemness by LIMP-2. GSEA of the TCGA-HNSCC dataset showed enrichment of the Wnt signaling pathway and pluripotency in LIMP-2^high^ expression subgroup compared to LIMP-2^low^ expression subgroup (Fig. 6a). We further analyzed both TCGA and GSE41613 databases for genes positively associated with LIMP-2 (top 1 000), of which 353 genes were indicated in both datasets. GO enrichment analysis results of these genes include lysosome, EMT and Wnt pathways (Fig. 6b, c). Further analysis showed that LIMP-2 was significantly positively related to Wnt pathway-related genes in a variety of tumors (Figs. S7a, S8).

Bioinformatics analysis guided us to examine whether Wnt signaling might be involved in maintaining the LIMP-2-induced CSC phenotype. We subsequently confirmed that silencing LIMP-2 inhibited the translocation of β-catenin from cytosol into nucleus and increased the expression of GSK3β (Fig. 6d). Western blot analysis demonstrated that knockdown of LIMP-2 inhibited the expression of β-catenin and its related downstream genes (Cyclin D1, c-Myc, N-cadherin, and vimentin) in 4MOSC2 cells, which was confirmed by the overexpression of LIMP-2 in 4MOSC1 cells (Figs. 6e, S7b).

To explore the role of the Wnt pathway in the LIMP-2-mediated malignant phenotype, we further knocked down β-catenin in LIMP-2-overexpressing 4MOSC1 cells. Knockdown of β-catenin significantly inhibited the enhanced expression of CSC-related markers induced by LIMP-2 overexpression (Fig. S7c, d). Similar results were observed in colony formation and tumor sphere formation assays (Fig. S7e–g). The knockdown of LIMP-2 contributed to reduced expression of β-catenin as well as elevated expression of GSK3β compared to the shNC group in 4MOSC2 orthotopic HNSCC mouse models (Fig. 6f). Consistently, patient specimens with low LIMP-2 expression tended to have lower β-catenin expression but higher GSK3β expression in our patient cohort (Fig. 6g).

Altogether, these results suggest that LIMP-2 regulates the stemness potential of HNSCC cells by activating GSK3β/β-catenin signaling.

### LIMP-2 degrades GSK3β in an autophagy-dependent manner

In the condition of Wnt/β-catenin signaling activation, GSK3β is sequestered within the lumen of late endosomes and degraded by lysosomes.^7^ And β-catenin is translocated from cytosol into nucleus and promotes the transcription of Wnt-target genes. Recent findings suggest that autophagy is involved in the degradation of GSK3β to maintain high expression of β-catenin, which implies the possible relationship between autophagy and Wnt signaling pathways.^26,27^

**Fig. 7:**
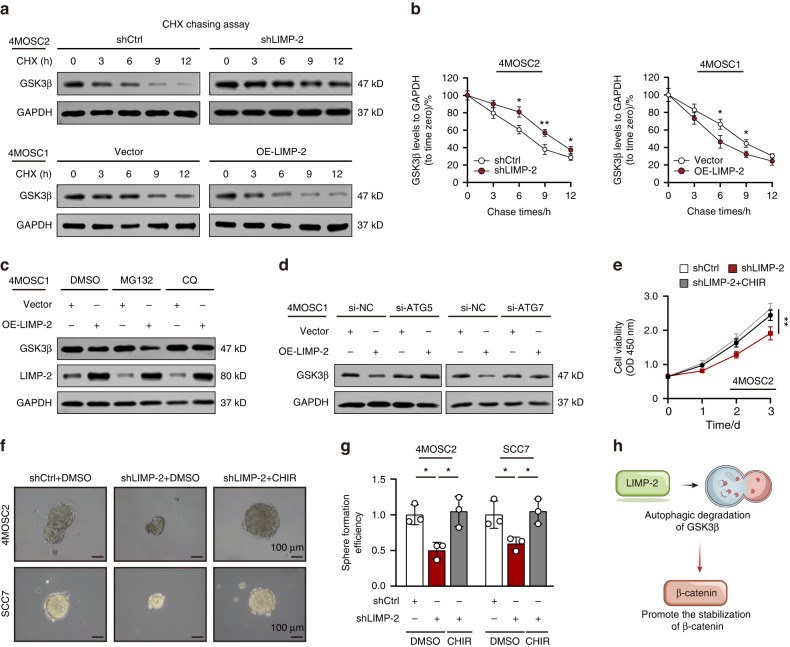
LIMP-2 degrades GSK3β in an autophagy-dependent manner. **a**, **b** Representative western blot results of GSK3β in shLIMP-2 4MOSC2 cells and OE-LIMP-2 4MOSC1 cells treated with 50 μg/ml cycloheximide (CHX). **c** Western blot results of GSK3β in control (empty vector) or LIMP-2-overexpressing 4MOSC1 cells treated with DMSO, MG132, or CQ. **d** Representative western blot results of GSK3β in control or LIMP-2-overexpressing 4MOSC1 cells treated with si-NC, si-ATG5, and si-ATG7. **e** Proliferation of shLIMP-2 4MOSC2 cells treated with CHIR was examined by CCK-8 assay. **f**, **g** Sphere formation ability of shLIMP-2 cells treated with CHIR. **h** LIMP-2 activates Wnt/β-catenin signaling by driving a selective autophagic GSK3β degradation mechanism. ^*^P < 0.05, ^**^P < 0.01, ^***^P < 0.001, and ns represents no significance

Our previous results showed that overexpression of LIMP-2 induced decreased protein levels of GSK3β (Fig. 6d, e). However, no changes in GSK3β mRNA levels were observed in LIMP-2-overexpressing cells (Fig. S9a), suggesting that LIMP-2 regulates GSK3β at the protein level. Considering the functional role of LIMP-2 in the autophagy‒lysosome pathway, we inferred that LIMP-2 regulates GSK3β degradation. The cycloheximide (CHX) assay showed that GSK3β degradation was delayed in the presence of LIMP-2 knockdown. Conversely, the overexpression of LIMP-2 markedly reduced the half-life of GSK3β (Fig. 7a, b). These results demonstrate that LIMP-2 accelerated the degradation of GSK3β. Notably, LIMP-2 overexpression-mediated GSK3β degradation was blocked by CQ (a autophagy inhibitor), instead of MG132 (a proteasome inhibitor) (Figs. 7c and S9b). To exclude off-target effects, we further knocked down ATG5 and ATG7 by siRNA in 4MOSC1 cells. The results showed that knockdown of ATG5 and ATG7 restored GSK3β expression in LIMP2-overexpressing 4MOSC1 cells (Fig. 7d). In addition, pharmacological inhibition of GSK3β (CHIR) rescued nuclear β-catenin, which is reduced in shLIMP-2 4MOSC2 cells (Fig. S9c). The restoration of proliferation and sphere formation ability of shLIMP-2 cells treated with CHIR demonstrated that GSK3β is required for LIMP-2-mediated malignant phenotype of HNSCC (Figs. 7e–g and S9d). Together, these data demonstrated that LIMP-2 promotes Wnt/β-catenin signaling by enhancing the autophagic degradation of GSK3β (Fig. 7h).

### Knockdown of LIMP-2 enhances the PD-1 blockade efficiency

**Fig. 8:**
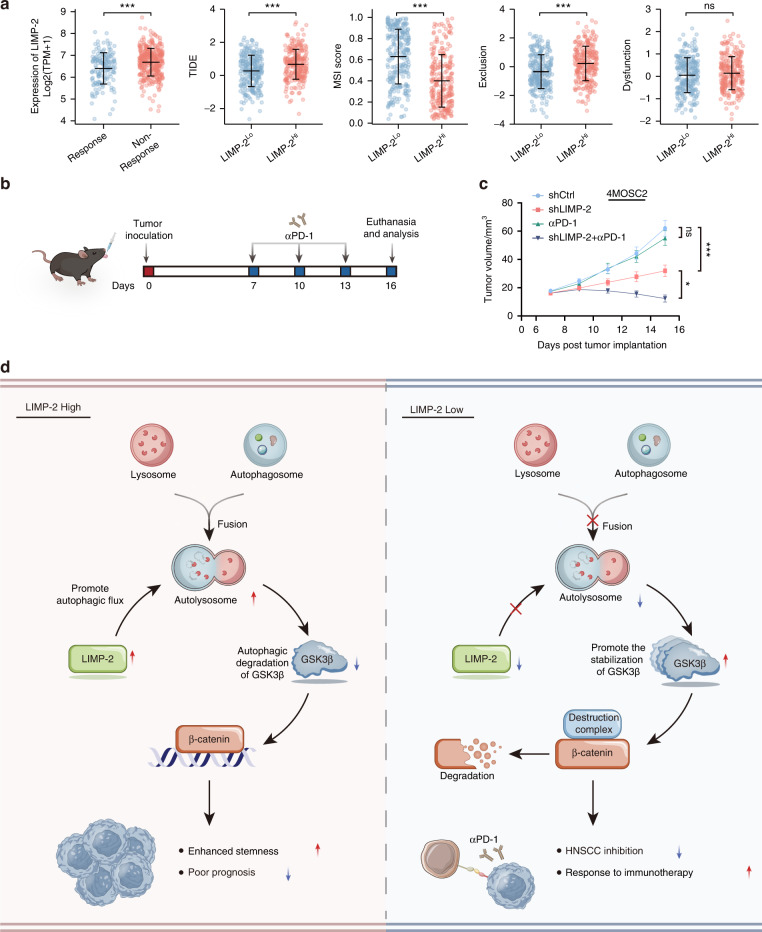
Knockdown of LIMP-2 improves the response to immunotherapy. **a** The TIDE algorithm was used to assess the effect of LIMP-2 on the potential efficacy of immunotherapy in the TCGA-HNSCC database. TIDE, MSI, and T-cell exclusion and dysfunction scores in subgroups with different expression levels of LIMP-2. Statistical differences were evaluated using the Wilcoxon test. **b** Schematic description of the experimental design used to establish the animal model. Orthotopic HNSCC model formation with 4MOSC2 cells (shNC or shLIMP-2), followed by treatment with an intraperitoneal injection of IgG2a isotype control antibody or αPD-1 antibody. **c** Tumor growth curves of different treatment groups. **d** Schematic diagram of the mechanisms by which LIMP-2 upregulation promotes HNSCC progression and CSC properties. The study revealed that LIMP-2 is involved in HNSCC progression and is an independent risk factor for poor survival in HNSCC patients. Mechanistically, LIMP-2 promotes autophagic degradation of GSK3β, which in turn contributes to nuclear translocation of β-catenin and increased expression of downstream target genes. Finally, activation of the Wnt/β-catenin pathway results in HNSCC progression and enhanced an CSC phenotype. All results were calculated in at least 3 independent experiments. ^*^p < 0.05, ^**^p < 0.01, ^***^p < 0.001, and ns represents no significance

Recent studies suggest that targeting CSCs may provide an exciting avenue to enhance the effectiveness of immunotherapy.^25^ To further assess the impact of LIMP-2 on the potential efficacy of immunotherapy, we evaluated the TIDE algorithm using the TCGA-HNSCC dataset (Fig. 8a). Higher TIDE prediction scores represent poorer immune checkpoint inhibitor (ICI) efficacy, meaning that patients are more likely to be resistant to ICI therapy.^28^ In our results, the TIDE scores in the LIMP-2 ^low^ expression subgroup were significantly lower than those in the LIMP-2 ^high^ expression subgroup, suggesting that patients with low LIMP-2 expression are prone to benefit from ICI treatment than patients with high LIMP-2 expression. In addition, we found higher microsatellite instability (MSI) scores and lower T-cell exclusion scores in the LIMP-2 ^low^ expression subgroup. However, T-cell dysfunction scores had no difference in the two subgroups (Fig. 8a). Subsequently, we evaluated the immune aspects of LIMP-2 in the tumor microenvironment (TME). The TCGA dataset and our TMA results showed that LIMP-2 was generally positively correlated with immune checkpoints (e.g., PD-L1, CD47, and B7-H3) in a variety of cancers, including HNSCC (Fig. S10a, b). Furthermore, we found that infiltrating macrophages were more abundant in the LIMP-2 ^high^ subgroup, while CD8^+^ T cells, follicular helper T cells, memory B cells, neutrophils, and plasma cells were more abundant in the LIMP-2 ^low^ subgroup (Fig. S10c). On this basis, we hypothesized that high levels of LIMP-2 expression in HNSCC may be associated with poor response to ICI treatment.

The 4MOSC2 model tends to develop lymph node metastasis and is prone to resistance to ICI therapy, making it suitable for investigating the mechanisms of immunotherapy resistance.^23^ We further used the 4MOSC2 model to test the potential combination efficacy of LIMP-2 knockdown with αPD-1 therapy (Fig. 8b). Although parental 4MOSC2 tumors were resistant to αPD-1 treatment, shLIMP-2 4MOSC2 tumors were significantly inhibited upon αPD-1 treatment (Figs. 8c and S11a, b). These findings demonstrate that targeting LIMP-2 can overcome αPD-1 treatment resistance and improve the efficacy of immunotherapy. Therefore, LIMP-2 may be a reliable biomarker for predicting patient response to PD-1 blockade therapy to provide a precise guide for clinical interference (Fig. 8d).

## Discussion

Currently, the treatment of HNSCC still faces the challenge of a high recurrence rate and high mortality, which is closely related to the biological characteristics of CSCs.^29–31^ Therefore, understanding the biological characteristics of CSCs and their potential drug targets is important for the diagnosis and treatment of HNSCC. The study revealed that LIMP-2 was involved in HNSCC progression and correlated with pathological stage, lymph node metastasis, gender, and patient prognosis. In terms of function, we identified a novel role for LIMP-2 in promoting HNSCC stemness. Further findings demonstrated that LIMP-2 promotes the CSC properties of HNSCC cells through the degradation of GSK3β via autophagy. These findings demonstrated that the LIMP-2/GSK3β/β-catenin axis promotes the malignant phenotype of HNSCC.

LIMP-2 is a type III glycoprotein located mainly on the restriction membrane of lysosomes and late endosomes.^14^ Lysosomal membrane proteins are widely involved in biological processes such as autophagosome-lysosome fusion and cholesterol transport. Past studies have found that lack of LIMP-2 but not LAMP-1 leads to impaired late endosome-lysosome fusion with phagosomes in mouse macrophages and therefore increased susceptibility to *Listeria monocytogenes* infection.^32^ In addition, mutations resulting in a functional deficiency of LIMP-2 did not affect lysosome formation but resulted in the inability of B lymphocytes to process autophagosomes.^33^ This implies a potential role of LIMP-2 in promoting the fusion of autophagosomes and lysosomes. Consistent with our hypothesis, our results found that the accumulation of autophagosomes is increased in cancer cells after LIMP-2 knockdown and that autophagic flux is impaired. These data suggest that LIMP-2 is involved in the formation of autolysosomes, which in turn promotes autophagic flux in HNSCC.

The plasticity of CSCs confers them the ability to adapt to therapeutic interference and the changing biological stresses of the TME throughout tumor evolution.^5,34^ There is growing evidence that autophagy is an essential regulator of maintaining plasticity and therapeutic resistance in CSCs.^25^ For example, AMBRA1 expression is dependent on c-Myc levels and induces stem cell potential, proliferation, and migration in medulloblastoma by affecting autophagy and STAT3 signaling.^35^ Furthermore, autophagy can maintain the proliferative potential and chemoresistance of CSCs through the nonclassical FOXO3/SOX2 axis in HNSCC.^13^ Collectively, these findings suggest that targeting autophagy can inhibit metastasis and CSC properties in a variety of cancers, including HNSCC. In our study, we found that elevated LIMP-2 is required to maintain the CSC phenotype in HNSCC. The use of the autophagy inhibitor CQ significantly inhibited CSC sphere formation, proliferation, and clone formation ability, suggesting that autophagy is involved in LIMP-2-induced malignant phenotypes in HNSCC.

Wnt pathway activation is induced by LIMP-2, and manifested by the nuclear translocation of β-catenin and increased expression of its target downstream genes (c-Myc, Cyclin D1). Our previous studies demonstrated that interference with CMTM6 inhibits the CSC properties of HNSCC and the TGF-β-induced EMT process by regulating the Wnt pathway.^36^ These results are encouraging and suggest that the classical Wnt/β-catenin pathway determines the differentiation fate and self-renewal potential of CSCs. Wnt signaling has been reported to induce cytosolic GSK3β sequestration into multivesicular endosomes fused with lysosomes and to inhibit GSK3β activity.^7,37^ Further studies revealed that autophagic activity is required for GSK3β segregation into late endosomes in adipogenesis.^27^ Considering the role of multivesicular endosomes/bodied in the degradation of autophagosomes, degradation may be the ultimate fate of Wnt-induced GSK3β segregation. A recent study showed that the autophagy receptor optineurin promotes Wnt signaling pathway-mediated myogenesis by directly interacting with GSK3β physically and targeting GSK3β for autophagic degradation.^26^ This phenomenon broadens the understanding of the autophagy-regulated Wnt pathway and reveals that GSK3β acts as a substrate for autophagy. Consistent with this, we found that LIMP-2 activates the Wnt pathway by promoting an autophagy-dependent GSK3β degradation mechanism to promote the proliferation and stemness of HNSCC. These results highlight the close connection between the CSC biology, autophagy, and the Wnt pathway in HNSCC. Interestingly, the relationship between autophagy and Wnt pathway is influenced by different cancer types and stages, with conflicting results. For example, autophagy can promote selective degradation of DVL2, leading to ubiquitination of β-catenin, which inhibits the Wnt signaling pathway.^38^ In this regard, autophagy is a negative modulator of Wnt pathway. In addition, autophagy activates the Wnt pathway by inhibiting GSK3β in myoblasts.^26,39^ Our findings demonstrated that the autophagy-mediated LIMP-2-GSK3β axis plays an active role in the Wnt signaling pathway in HNSCC.

Mounting evidence suggests a connection between acquisition of CSC characteristics and immunotherapy resistance in multiple carcinoma types including HNSCC.^40^ Indeed, targeting CSCs, such as inhibiting BMI1, and CD276, is effective in improving immunotherapy efficacy, suggesting that CSCs may be responsible for immune evasion in HNSCC.^5,6,29^ As expected, we found that patients with low LIMP-2 expression were prone to benefit from ICI therapy and further validated this finding with in vivo experiments, which may be related to the function of LIMP-2 to maintain CSC-like properties. This result implies that LIMP-2 represents a potential therapeutic target with clinical utility for HNSCC patients, likely in combination.

In summary, our findings suggest that LIMP-2 is an independent poor prognostic factor for HNSCC. Elevated LIMP-2 is required to maintain the CSC phenotype and implies increased immunotherapy resistance in HNSCC. This study revealed compelling evidence for the critical role of LIMP-2 as a potential therapeutic target for diagnosis and therapy of HNSCC.

## Materials and methods

Details are provided in Supplementary information.

### Tissue specimens

The study was approved in accordance with the institutional guidelines (2016LUNSHENZI62). In this study, 210 primary HNSCC samples, 69 oral epithelial dysplasia samples, and 42 normal mucosal samples were included in the HNSCC tissue cohort.^36^ All human HNSCC samples were obtained from the Hospital of Stomatology of Wuhan University (more details of the pathological characteristics are shown in Table S1). Time inducible tissue specific *Tgfbr1/Pten* 2cKO mice (*K14‐Cre*^*ERtam+/−*^; *Tgfbr1*^*flox/flox*^; *Pten*^*flox/flox*^) were kindly provided by Dr. Ashok B. Kulkarni (National Institute of Dental and Craniofacial Research, USA) with material transfer agreement (NIH T-2012-1735) and United States Department of Agriculture certification (VA-12-4122R).^22^

### Cell lines and cell culture

4MOSC1 and 4MOSC2 cells were derived from 4NQO-induced tongue tumors isolated from female C57BL/6 mice and maintained in keratinocyte serum-free medium (Gibco, USA).^23^ Complied with a material transfer agreement (SD-2017-202), 4MOSC1 and 4MOSC2 cells were generous gifts from Prof. J. Silvio Gutkind (University of California San Diego, USA). The mouse HNSCC cell line SCC7 was gifted by Prof. Qianming Chen (Sichuan University, China) and was maintained in RPMI 1640 medium. Mycoplasma contamination detection was performed annually by PCR. For any experiment, cells were grown for no more than 15 generations in total.

### Cell proliferation assay

Cell viability, colony formation, and EdU assays were carried out according to the conventional protocol.^36^ In brief, the analysis of cell viability in 4MOSC1, 4MOSC2, and SCC7 was used by the CCK-8 assay (Dojindo, Japan). HNSCC cells (500/well) were cultured in six-well plates for 10 days in the colony formation assay. And colony analysis was then performed using ImageJ software. For EdU assays, proliferating HNSCC cells were stained using the EdU assay kit (Beyotime, China) and visualized by fluorescence microscopy.

### Sphere formation assay

HNSCC single cells were resuspended in sphere formation medium (DMEM-F12 + 1% B27 supplement + 20 ng·mL^−1^ bFGF + 20 ng·mL^−1^ EGF + 1% N2 supplement) and inoculated in ultralow attachment 6-well plates (1 000 cells per well, Corning, USA). The size and number of tumor spheres formed were counted according to the conventional protocol.

### Cell line transfection

For knocking down the expression of LIMP-2, the specific shRNA against LIMP-2 (shLIMP-2) and control shRNA (shCtrl) were constructed and packaged by Ubigene Biosciences (Guangzhou, China). The specific targeting sequence was as follows: shLIMP-2 (mouse): 5’-GGGTCTATGGATGAGGGAA-3’. The 4MOSC2 and SCC7 cells were infected with polybrene and lentiviral supernatant and then screened with 4 μg·mL^−1^ puromycin (Sigma‒Aldrich, USA). The Protein levels were verified using Western blot.

Full-length mouse LIMP-2 complementary DNA was PCR amplified and cloned into the pcDNA3.1 plasmid vector (Tsingke Biotechnology Co. Ltd., China). β-catenin-siRNA (5ʹ-UACAUCAUUUGUAUUCUGCTT-3ʹ), ATG5-siRNA (5ʹ- GCGGUUGAGGCUCACUUUATT-3ʹ), and ATG7-siRNA (5ʹ-GCUAGAGACGUGACACAUATT-3ʹ) were from GenePharma (Suzhou, China). The mRFP-GFP-LC3 plasmid were kindly donated by Dr. T Yoshimori and distributed by Addgene. All the plasmids were transfected into HNSCC cells using Lipofectamine™ 3000 following protocol (Invitrogen, USA).

### Western blot and real-time PCR analysis

For western blot assays, RIPA buffer containing protease inhibitors and phosphatase inhibitors (Roche, UK) was added to extract proteins from HNSCC tissues and cells. Nucleoprotein extraction kit (Beyotime, China) was used to isolate nuclear proteins. A list of primary antibodies used for western blot assay is provided in the supplementary files. For real-time PCR, total RNA was purified from HNSCC cells with an RNA isolation kit (Axygen, USA) as directed by the manufacturer. The cDNA was reverse-transcribed by the Reverse Transcription System (Vazyme, China) and was used for quantitative PCR amplification by the Bio-Rad-CFX96 system. The primer sequences are shown as follows: GSK3β (F: 5ʹ- AAGCGATTTAAGAACCGAGAGC -3ʹ, R: 5ʹ- AGAAATACCGCAGTCGGACTAT-3ʹ).

### Immunohistochemistry (IHC) and immunofluorescence

IHC staining was performed and analyzed as described previously.^36^ A list of primary antibodies used for immunohistochemistry is provided in the supplementary files. All slides were scanned and quantified by a Pannoramic Midi (3DHISTECH). The histoscore was normalize to a minimum value of 0 and a maximum value of 300. For immunofluorescence staining, HNSCC cells were incubated with primary antibodies against the following markers: CD44 (Proteintech), ALDH1A1 (Proteintech), β-catenin (Abmart), and anti-GSK3β (Abcam), followed by fluorescence-conjugated secondary antibodies (Dylight 488/594, Abbkine) for imaging. The fluorescence signals were visualized by a confocal microscope (FV1200, Olympus Life Science).

### Flow cytometry

To detect CD44 expression on the cell surface, 4MOSC2 and SCC7 single cell suspensions were prepared and the cells were then incubated with PE-conjugated anti-CD44 antibody (Cell Signaling Technology). ALDH activity was detected with the ALDEFLUOR^TM^ kit (STEMCELL Technologies) as described previously.^36^ For each sample, one-half of the cells had added diethylaminobenzaldehyde (DEAB) to define negative gates. Cell apoptosis rate in each sample was evaluated with the annexin V-FITC/ PI kit following the manufacturer’s protocol (EBioscience, USA).

### Animal experiment

The care and experimental procedures of all animals were approved by the Animal Ethics Committee of the School and Hospital of Stomatology of Wuhan University (S07921080D). 4MOSC2 (1.0 × 10^6^) cells were grafted into the dorsum linguae of female C57BL/6 mice to establish orthotopic HNSCC mouse models. SCC7 cells (1.0 × 10^6^) were administered subcutaneously into the right hind region of female C3H/HeNCr MTV (C3H) mouse to establish an ectopic allograft model of HNSCC. For in vivo PD-1 blockade therapy assays, orthotopic HNSCC mouse models were constructed. After the tumor appeared, the mice were administered intraperitoneally (i.p.) with IgG2a isotype control antibody (BE0086; Bio X Cell) or αPD-1 antibody (BE0146; Bio X Cell) every three days. All experimental mice were strictly observed and euthanized on day 16 after tumor injection. Calculation of tumor volume (mm^3^) was applied: width^2^ × length × 1/2.

### Bioinformatics analysis

The publicly-available transcriptomic data for 546 HNSCC samples (including 502 HNSCC cases and 44 normal cases) were downloaded from The Cancer Genome Atlas (TCGA) database. The RNA-seq data for 97 HNSCC cases (GSE41613) were available from the GEO database. In our study, we identified genes involved in autophagy‒lysosome biological processes (*n* = 135) based on confirmed lysosome proteomics, lysosomal disorders-related datasets, and autophagy-interacting genes (Table S2).^20^ Functional enrichment analysis of the genes related to the autophagy‒lysosome pathway was performed. GO and KEGG analyses were executed with the Bioconductor package “clusterProfiler” (Fig. S1A). Subsequently, we screened the TCGA-HNSCC and GSE41613 databases for genes involved in unfavorable overall survival (OS) by Cox regression analysis (*P* < 0.05, HR > 1, median histoscore as cutoff value). LIMP-2 was shown in the set of genes associated with the autophagy‒lysosome pathway and in two independent sets of genes associated with poor prognosis in HNSCC. The protein‒protein interaction network of LIMP-2 and genes related to the autophagy‒lysosome pathway was visualized by using the STRING database (Fig. S1B). GSEA and single-sample GSEA (ssGSEA) analyses were executed to conduct signaling pathway analysis. Procedure details of the bioinformatics analysis are available in the supporting documentation.

### Statistical analysis

All statistical analyses were performed with GraphPad Prism 9 and R 3.6.3 software in at least 3 independent experiments. Student’s *t*-test and one-way ANOVA test were used to analyze differences between two and multiple groups, respectively. Categorical variables were analyzed by the chi-square (χ^2^) test. TIDE score and proportions of TME cells between groups were compared using the Wilcoxon test. Spearman’s correlation analysis was used to perform the correlation analysis. Survival analysis was performed using univariate Cox regression. All error bar values represent the standard deviation (SD) or standard error of mean (SEM). In all types of statistical analysis, *p* < 0.05 was considered statistically significant.

## Supplementary information

Supplementary Materials and Methods

Supplementary Figures

Supplementary Tables

## Data Availability

All data in this study are available upon reasonable request to the authors.
