# Author Correction: LIMP-2 enhances cancer stem-like cell properties by promoting autophagy-induced GSK3β degradation in head and neck squamous cell carcinoma

**DOI:** 10.1038/s41368-025-00358-8

**Published:** 2025-03-28

**Authors:** Yuantong Liu, Shujin Li, Shuo Wang, Qichao Yang, Zhizhong Wu, Mengjie Zhang, Lei Chen, Zhijun Sun

**Affiliations:** 1 The State Key Laboratory Breeding Base of Basic Science of Stomatology (Hubei-MOST) & Key Laboratory for Oral Biomedicine Ministry of Education, School and Hospital of Stomatology, Wuhan University, Wuhan, China; 2 Department of Oral Maxillofacial-Head Neck Oncology, School and Hospital of Stomatology, Wuhan University, Wuhan, China

**Keywords:** Oral cancer, Cancer stem cells, Autophagy, Mechanisms of disease

Correction to: *International Journal of Oral Science* **15**, 24 (2023); 10.1038/s41368-023-00229-0, published online 08 June 2023

Following publication of the original article,^1^ the authors reported an error in Supplementary Fig. 7f. Specifically, the image representing the OE-LIMP-2+si-β-catenin experimental group in Supplementary Figure 7f was corrected. The corrected Supplementary Fig. 7f is presented below. All authors have confirmed that this error does not affect the original conclusions of the study. The supplementary file of original article has been updated. We apologize for this error.

The corrected Supplementary Fig. 7 was:

**Figure 1:**
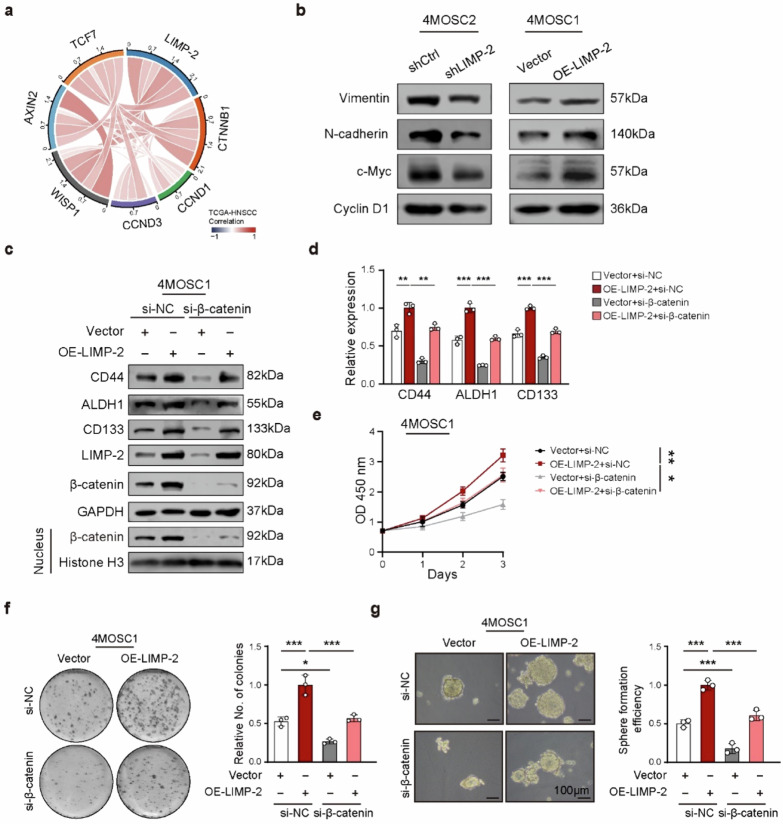
**Supplementary Fig. 7.** **a** Spearman’s correlation between LIMP-2 expression with Wnt pathway-related genes in TCGA-HNSCC dataset. **b** Western blot results and quantification of Wnt pathway-related genes in indicated cell lines. **c**, **d** Western blot assays indicated that β-catenin knockdown abolished the promoting effects of LIMP-2 on the expression of CSC-related markers. Histone H3 was loaded as a nuclear marker. The proliferation of vector and OE-LIMP-2 4MOSC1 cells treated with siNC or siβ-catenin was examined by CCK-8 assay **e** and colony formation assay **f**. **g** The stemness of 4MOSC1 cells was examined by tumor sphere formation assay. All results were calculated in at least three independent experiments and expressed as mean ± SD. ^*^p < 0.05, ^**^p < 0.01, ^***^p < 0.001

Originally published Supplementary Fig. 7:

**Figure 2:**
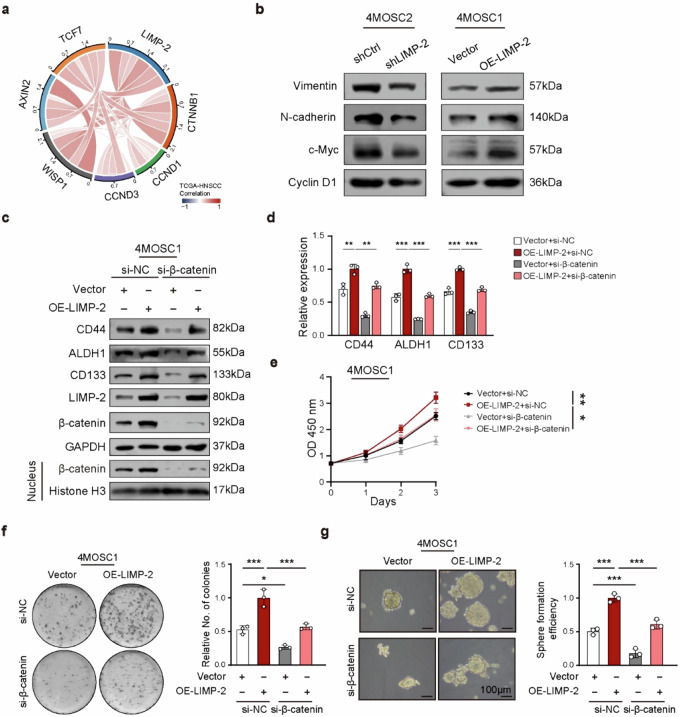
**Supplementary Fig. 7.** **a** Spearman’s correlation between LIMP-2 expression with Wnt pathway-related genes in TCGA-HNSCC dataset. **b** Western blot results and quantification of Wnt pathway-related genes in indicated cell lines. **c**, **d** Western blot assays indicated that β-catenin knockdown abolished the promoting effects of LIMP-2 on the expression of CSC-related markers. Histone H3 was loaded as a nuclear marker. The proliferation of vector and OE-LIMP-2 4MOSC1 cells treated with siNC or siβ-catenin was examined by CCK-8 assay **e** and colony formation assay **f**. **g** The stemness of 4MOSC1 cells was examined by tumor sphere formation assay. All results were calculated in at least three independent experiments and expressed as mean ± SD. ^*^p < 0.05, ^**^p < 0.01, ^***^p < 0.001

In addition, the Ithenticate’s Plagiarism Detection was published as a Supplementary file and has been removed.

The original article^1^ has been updated.

## References

[1] Liu, Y. et al. LIMP-2 enhances cancer stem-like cell properties by promoting autophagy-induced GSK3β degradation in head and neck squamous cell carcinoma. *Int J Oral Sci* **15**, 24 10.1038/s41368-023-00229-0 (2023). 37291150 10.1038/s41368-023-00229-0 PMC10250453

