# Supplementary material for: LIMP-2 enhances cancer stem-like cell properties by promoting autophagy-induced GSK3β degradation in head and neck squamous cell carcinoma
Source: Int J Oral Sci. 2023 Jun 8;15:24. doi: 10.1038/s41368-023-00229-0 (PMC10250453; doi:10.1038/s41368-023-00229-0)
Supplement: Supplementary file 1 — Supplementary Materials and Methods [file 41368_2023_229_MOESM1_ESM.docx]

**LIMP-2 enhances cancer stem-like cell properties by promoting autophagy-induced GSK3β degradation in head and neck squamous cell carcinoma**

Yuan-Tong Liu^1^, Shu-Jin Li^1^, Shuo Wang^1^, Qi-Chao Yang^1^, Zhi-Zhong Wu^1^, Meng-Jie Zhang^1^, Lei Chen^1^, Zhi-Jun Sun^1, 2, *^

^1^ The State Key Laboratory Breeding Base of Basic Science of Stomatology (Hubei-MOST) & Key Laboratory for Oral Biomedicine Ministry of Education, School and Hospital of Stomatology, Wuhan University, Wuhan, China.

^2^ Department of Oral Maxillofacial-Head Neck Oncology, School and Hospital of Stomatology, Wuhan University, Wuhan, China.

*Corresponding author:

Zhijun Sun, School and Hospital of Stomatology, Wuhan University, Wuhan 430079, China; Email: sunzj@whu.edu.cn.

The authors declare no potential conflicts of interest.

**Supplementary materials and methods**

**Tissue specimens**

Full ethical approval was granted by the School and Stomatology of Wuhan University Medical Ethics Committee (2016LUNSHENZI62). All human HNSCC specimens were obtained from the Hospital of Stomatology of Wuhan University from 2011 to 2016. The tissue microarrays (TMAs) were constructed from 42 normal oral mucosae, 69 oral epithelial dysplasia, 210 tumor tissues from primary HNSCC patients, and 57 metastatic lymph node tissues from the primary HNSCC patients. The clinical features, including tumor-node-metastasis (TNM) staging, histological grade, and overall survival were available for all cases. The clinical and pathological grade analysis was performed according to the guidelines of the Union for International Cancer Control (UICC 2002) and the World Health Organization grading scheme (more details of the pathological characteristics are shown in Table S1) ^1^. For all collected tissue samples, H&E slides were reviewed by two independent pathologists. The clinicopathologic characteristics and follow-up data for these patients were retrieved from the electronic medical records.

The details of time inducible tissue specific *Tgfbr1/Pten* 2cKO mice (*K14‐Cre^ERtam+/−^*; *Tgfbr1^flox/flox^*; *Pten^flox/flox^*) were maintained and genotyped according to published protocols ^2^. All the mice were bred in the FVBN/CD1/129/C57 mixed background. The mouse tissue was gifted by Dr. Ashok B. Kulkarni (National Institute of Dental and Craniofacial Research, USA) with a material transfer agreement (NIH T-2012-1735) and United States Department of Agriculture certification (VA-12-4122R).

**Cell lines and cell culture**

4MOSC1 and 4MOSC2 cells were established from a murine 4NQO-induced tongue tumor arising in a female C57BL/6 mouse and cultured in keratinocyte serum-free medium (Gibco, USA) ^3, 4^. 4MOSC1 and 4MOSC2 cells were generous gifts from Prof. J. Silvio Gutkind (University of California San Diego, USA) with a material transfer agreement (SD-2017-202). The mouse HNSCC cell line SCC7 was gifted by Prof. Qianming Chen (Sichuan University, China) and was cultured in RPMI 1640 medium. Mycoplasma contamination detection was performed annually by PCR. For any experiment, cells were grown for no more than 15 generations in total.

**Antibodies and Reagents**

The following primary antibodies were used for western blot, immunohistochemistry (IHC), and immunofluorescence: Rabbit anti-LIMP-2 (ab176317), rabbit anti-LC3B (ab192890), rabbit anti-SQSTM1/p62 (ab109012), rabbit anti-ATG5 (ab108327), rabbit anti-ATG7 (ab133528), rabbit anti-GSK3β (ab32391), rabbit anti-Cyclin D1 (ab134175) were all from Abcam. Rabbit anti-CD44 (15675-1-AP), rabbit anti-ALDH1A1 (15910-1-AP), rabbit anti-CD133 (18470-1-AP), rabbit anti-BMI1 (10832-1-AP), and anti-GAPDH (HRP-60004) were bought from Proteintech (China). Mouse anti-β-Catenin (M24002) was from Abmart. Rabbit anti-Ki67 (#9129), rabbit cleaved caspase-3 (#9661), rabbit anti-SOX2 (#10652), rabbit anti-c-Myc (#5605), rabbit anti-Histone H3 (#4499), and anti-CD44-PE (#88151) were purchased from Cell Signaling Technology (CST). The chemical reagents used were obtained from MedChemExpress (USA), including chloroquine (HY-17589A), MG-132 (HY-13259), cycloheximide (HY-12320), and CHIR-99021 (HY-10182).

**Cell proliferation assay**

Cell viability, colony formation, and EdU assays were performed as described previously ^1^. In brief, 4MOSC1, 4MOSC2, and SCC7 cell viability was measured by the CCK-8 assay (Dojindo, Japan). Cancer cells at a density of 2000 cells per well in the corresponding medium were inoculated into 96-well plates for CCK-8 assays. At 24, 48, and 72 h, 20 μl CCK-8 was added to each well and incubated at 37°C for 2 hours. The absorbance was then measured at 450 nm using a BioTek plate reader (BioTek). For the colony formation assay, 500 cancer cells were cultured in six-well plates for 10 days. Cells were fixed using 4% paraformaldehyde and then stained using crystal violet dyes. Colonies’ numbers were counted by ImageJ. For the EdU assay, an EdU assay kit (Beyotime, China) was used to stain proliferating HNSCC cells according to the manufacturer's instructions. EdU-positive cells were visualized by fluorescence microscopy.

**Sphere formation assay**

HNSCC single cells were resuspended in sphere formation medium (DMEM-F12 + 1% N2 supplement + 1% B27 supplement + 20 ng/ml EGF + 20 ng/ml bFGF) and inoculated in ultralow attachment 6-well plates (1000 cells/well, Corning, USA). After culturing for 10 days, spheres larger than 100 μm in diameter were counted. The sphere formation efficiency was calculated as the number of spheres/number of seeded cells×100%, and that of control groups was set as 1.0.

**Cell line transfection**

For knocking down LIMP-2, one short hairpin RNA targeting LIMP-2 (shLIMP-2) and the scramble control short hairpin RNA (shCtrl) were constructed and packaged by Ubigene Biosciences (Guangzhou, China). The specific targeting sequence was as follows: shLIMP-2 (mouse): 5'-GGGTCTATGGATGAGGGAA-3'. Then, the 4MOSC2 and SCC7 cells were infected with 8 μg/ml polybrene and lentiviral supernatant. Twenty-four hours after transfection, the cells were screened with 4 μg/ml puromycin (Sigma‒Aldrich, USA).

Full-length mouse LIMP-2 complementary DNA was PCR amplified and cloned into the pcDNA3.1 plasmid vector according to the manufacturer’s instructions (Tsingke Biotechnology Co. Ltd., China). Negative control-siRNA（5′-UUCUCCGAACGUGUCACGUTT-3′）, β-catenin-siRNA (5ʹ-UACAUCAUUUGUAUUCUGCTT-3ʹ), ATG5-siRNA (5ʹ- GCGGUUGAGGCUCACUUUATT-3ʹ), and ATG7-siRNA (5ʹ-GCUAGAGACGUGACACAUATT-3ʹ) were from GenePharma (Suzhou, China). The tandem fluorescent-tagged LC3 construct plasmid (tfLC3, mRFP-GFP-LC3) was a generous gift from Dr. T Yoshimori and distributed by Addgene. All the plasmids were transfected into HNSCC cells using Lipofectamine™ 3000 according to the manufacturer’s protocol (Invitrogen, USA).

**Western blot and real-time PCR analysis**

To collect cell lysates, cells were washed with PBS and lysed with RIPA buffer supplemented with protease and phosphatase inhibitors (Roche, UK). Collected cells were lysed in SDS sample buffer, and boiled for 10 minutes. Cell lysates containing equal amounts of protein were separated by SDS‐PAGE and transferred to PVDF membranes (Millipore). The membranes were blocked in 5% nonfat milk in TBST (Tris‐buffered saline containing 0.1% Tween‐20) for 1 hour and then incubated with the primary antibodies at 4°C overnight and then exposed to appropriate secondary antibodies for 1 hour at room temperature. After being washed with TBST three times, the membranes were visualized using the ECL system (Thermo Fisher, USA). Nuclear proteins were isolated using a nucleoprotein extraction kit (Beyotime, China). The following primary antibodies were used for western blot analysis: anti-LIMP-2 antibody (1:1000, Abcam), anti-LC3B (1:2000, Abcam), anti-SQSTM1/p62 (1:10000, Abcam), anti-ATG5 (1:1000, Abcam), anti-ATG7 (1:10000, Abcam), anti-CD44 (1:4000, Proteintech), anti-ALDH1A1 (1:1000, Proteintech), anti-SOX2 (1:1000, CST), anti-CD133 (1:2000, Proteintech), anti-cleaved caspase 3 (1:1000, CST), anti-β-catenin (1:1000, Abmart), anti-c-Myc (1:1000, CST), anti-GSK3β (1:5000, Abcam), anti-Cyclin D1 (1:10000, Abcam) ,anti-Histone H3 (1:2000, CST), and GAPDH (1:5000, Proteintech).

Real-time PCR was performed as described in our previous study ^5^. Total RNA was purified from HNSCC cells using an RNA isolation kit (Axygen, USA) according to the manufacturer's instructions. The cDNA was reverse-transcribed from total RNA using the Reverse Transcription System (Vazyme, China) and was used for quantitative PCR amplification by Bio-Rad-CFX96 real-time PCR system. The primer sequences are shown as follows: GSK3β (F: 5ʹ- AAGCGATTTAAGAACCGAGAGC -3ʹ, R: 5ʹ- AGAAATACCGCAGTCGGACTAT-3ʹ).

**Immunohistochemistry (IHC) and** **immunofluorescence**

IHC staining of paraffin-embedded human or mouse tumor sections was performed according to standard protocols ^1^. Sections were deparaffinized, rehydrated, subjected to antigen retrieval, and blocked with hydrogen dioxide and goat serum, followed by incubating in primary antibodies overnight at 4 °C. The primary antibodies used were listed as follows: anti-LIMP-2 (1:100, Abcam), anti-Ki67 (1:1000, CST), anti-CD44 (1:500, Proteintech), anti-ALDH1A1 (1:100, Proteintech), anti-SQSTM1/p62 (1:500, Abcam), anti-β-catenin (1:200, Abmart), and anti-GSK3β (1:100, Abcam). HRP-conjugated secondary antibodies (undiluted, anti-rabbit/anti-mouse, CST, Cat 8114/8125) were then used. The signals were detected by the DAB substrate kit (CST). All slides were scanned and quantified by a Pannoramic Midi (3DHISTECH). The histoscore of staining was calculated as a percentage of positive cells using the formula (total intensity of strong positive) ×3 + (total intensity of positive) × 2 + (total intensity of weak positive) × 1. The histoscore was normalized to 0–300.

Immunofluorescence staining was performed as previously described ^1^. Cells were cultured in the confocal dish for 48–72 h and then washed with PBS three times and fixed in 4% paraformaldehyde for 30 min, permeabilized with 0.5% Triton X-100, followed by blocking with goat serum. Then cells were subjected to staining with anti-CD44 antibody (1:100, Proteintech), anti-ALDH1A1 (1:100, Proteintech), anti-β-catenin (1:100, Abmart), or anti-GSK3β (1:100, Abcam) antibody, followed by fluorescence-conjugated secondary antibodies (Dylight 488/594, Abbkine) for imaging. The cells were then counterstained with DAPI as a nuclear indicator and visualized by a confocal microscope (FV1200, Olympus Life Science).

**Transmission electron microscopy (TEM) analysis**

The HNSCC cells were harvested and fixed in glutaraldehyde for 2 h. Then, the cells were postfixed in 1% osmic acid for 2 h and dehydrated using graded ethanol (50–100%). Subsequently, the cells were embedded in Spur resin and cut into ultrathin sections (60 μm). Finally, the sections were stained with uranyl acetate and lead citrate. TEM (HT7700, Hitachi, Japan) was used to acquire images of autophagosomes in cells.

**Flow cytometry**

For the detection of CD44 expression on the cell surface, PE-conjugated anti-CD44 antibody (1:100, CST) was used to stain the cells after different treatments. For control, IgG isotype control (1:100, CST) was used. The ALDEFLUOR^TM^ Kit (STEMCELL Technologies) was used to analyze aldehyde dehydrogenase (ALDH) enzyme activity. A total of 10^6^ cells/ml in ALDEFLUOR^TM^ Assay Buffer were incubated with ALDEFLUOR^TM^ reagent, and diethylaminobenzaldehyde (DEAB) was added to each sample as a negative control. For the apoptosis detection assay, cells were digested with EDTA-free trypsin and washed with PBS buffer followed by detection with an annexin V-FITC/PI apoptosis kit (EBioscience, USA). All samples were assayed using flow cytometry (Beckman Coulter) and were then analyzed using FlowJo software (Treestar).

**Animal experiment**

All animal studies were approved by the Animal Ethics Committee of the School and Hospital of Stomatology of Wuhan University (S07921080D). All animal experimental procedures were performed in accordance with the Regulations for the Administration of Affairs Concerning Experimental Animals approved by the State Council of the People’s Republic of China. All mice were housed under specific pathogen-free (SPF) conditions (temperature ~22 °C, humidity ~50%) with a 12/12 h dark/light cycle. 6- to 8-week-old female C57BL/6 mice and C3H/HeNCr MTV (C3H) mice were purchased from Beijing Vital River Laboratory Animal Technology. 4MOSC2 (1.0×10^6^) cells were transplanted into the dorsum linguae of C57BL/6 mice to establish orthotopic HNSCC mouse models. SCC7 cells (1.0×10^6^) were injected subcutaneously into the right hind flank of each C3H mouse to establish an ectopic allograft model of HNSCC. The body weight and tumor volume of each mouse were recorded every 2 days, and tumor volume (mm^3^) was calculated as follows: width^2^ × length × 1/2. All mice were euthanized on day 16 after inoculation, and tumor samples were collected for immunohistochemistry and functional analysis.

For *in vivo* PD-1 blockade therapy assays, orthotopic HNSCC mouse models were constructed. After the tumor appeared, the mice were treated intraperitoneally (i.p.) with IgG2a isotype control antibody (10 mg/kg; BE0086; Bio X Cell) or α-PD-1 antibody (10 mg/kg; BE0146; Bio X Cell) every three days. The mice were strictly observed and tumor volumes were measured every two days. When the treatment experiment was complete, the mice were euthanized and dissected to obtain the tumors.

**Bioinformatics analysis**

The RNA-seq data for 546 HNSCC cases including 502 HNSCC tumor samples and 44 normal tissue samples were downloaded from The Cancer Genome Atlas (TCGA) database derived from the data portal (https://portal. gdc.cancer.gov/). The RNA expression data and survival information for 97 HNSCC samples (GSE41613) were downloaded from Gene Expression Omnibus (<https://www.ncbi.nlm.nih.gov/geo/>, GEO: GSE41613) ^6^. Procedure details are provided as follows.

**Genes** **related to the autophagy-lysosome pathway and gene screening**

In our study, we identified 135 genes from a comprehensive autophagy‒lysosome gene signature based on data from existing studies (Table S2) ^7^. These genes involve: (1) known lysosome proteomics, (2) autophagy interactome datasets, and (3) published lysosomal disease-related genes. Functional enrichment analysis of the genes related to the autophagy‒lysosome pathway was performed. Gene Ontology (GO) and Kyoto Encyclopedia of Genes and Genomes (KEGG) analyses were performed using the “clusterProfiler” package (Fig. S1a) ^8^. The bubble map was drawn using the “ggplot2” R package ^9^.

Univariate Cox regression analysis was used to screen the TCGA-HNSCC and GSE41613 databases for genes significantly related to overall survival (OS) (Table S3, S4). The bubble map and volcano plots were plotted using the R package ggplot2 ^9^. LIMP-2 was shown in the set of genes associated with the autophagy‒lysosome pathway and in two independent sets of genes associated with poor prognosis in HNSCC. The protein‒protein interaction network was visualized by using the STRING database (https://string-db.org/). Subsequently, the PPI network was visualized using Cytoscape software (Fig. S1b) ^10, 11^.

**Prognosis analysis**

HNSCC patients were divided into two groups according to the best cutoff and median cutoff values, respectively, based on the level of LIMP-2 expression. The surv_cutpoint function in the R Package “survminer” was introduced to determine the best cutoff value for dissecting the population. Overall survival of the expression of LIMP-2 was evaluated by the Kaplan–Meier survival curve. The hazard Ratio (HR) of death associated with LIMP-2 expression was estimated by the univariate Cox proportional hazards regression model first. Multivariate Cox model was then constructed to estimate the adjusted HR for LIMP-2 expression. Kaplan-Meier and Cox regression analyses were conducted to explore the influence of LIMP-2 on the prognosis of patients in HNSCC using the R package “survminer” and “survival” ^12^. *P* values less than 0.05 were considered statistically significant.

**Gene set enrichment analysis (GSEA) and single-sample GSEA (ssGSEA)**

In the signaling pathway analysis, HNSCC patients were first divided into two groups according to the median expression level of LIMP-2. Then, differential expression analysis was applied between the high and low expression groups (Table S5). Input genes for GSEA were sorted by their logFC values. Enrichment analysis to determine the signaling pathways in which the differentially expressed genes are involved was then carried out by using the GSEA method with the clusterProfiler package of R (FDR < 0.25 and *p* < 0.05) ^13, 14^.

H.all.v7.5.1.symbols.gmt (Hallmarks) was considered a predefined gene set and downloaded from the Molecular Signatures Database (MSigDB, http://software.broadinstitute.org/gsea/ msigdb). The hallmark scores were calculated with the transcriptome profiling data using ssGSEA algorithm (R package “GSVA”) ^15^. The correlation between hallmark scores and LIMP-2 expression was analyzed by Spearman’s correlation.

**Immune landscape analysis**

To predict the immunotherapy response in different LIMP-2 subgroups, tumor immune dysfunction and exclusion (TIDE) scores of 502 HNSCC samples were calculated with the algorithm provided by Jiang et al ^16^. Moreover, the TIDE score can evaluate two distinct mechanisms of tumor immunity: the dysfunction of tumor-infiltrating cytotoxic T lymphocytes (CTLs), and the exclusion of CTLs by immunosuppressive factors.

With transcriptome profiling data, the CIBERSORT algorithm was used to assess the abundance of 22 immune cell types ^17^. The correlation between the expression of immune checkpoint-related genes, autophagy-related genes, Wnt pathway-related genes, and expression of LIMP-2 was calculated by Spearman’s correlation analysis by the TIMER database (http://timer.comp-genomics.org/) (Table S6) ^18, 19^.

**Statistical analysis**

All statistical analyses were conducted using GraphPad Prism 8.0 and R 3.6.3 software. Two-tailed Student’s t test was applied for comparisons between two groups, and one-way ANOVA with Tukey’s multiple comparisons test was applied for multiple comparisons. Spearman’s correlation analysis was used to perform the correlation analysis. Categorical variables were analyzed by the chi-square (χ^2^) test. TIDE score and proportions of TME cells between groups were compared using the Wilcoxon test. Survival analysis was performed using univariate Cox regression. All results were calculated in at least three independent experiments. All error bar values represent the standard deviation (SD) or standard error of mean (SEM). In all types of statistical analysis, *p* values < 0.05 were considered statistically significant (^*^*p* < 0.05, ^**^*p* < 0.01, and ^***^*p* < 0.001).

**References**

1. Chen L, et al. Targeting CMTM6 Suppresses Stem Cell-Like Properties and Enhances Antitumor Immunity in Head and Neck Squamous Cell Carcinoma. Cancer Immunol. Res. **8**, 179-91 (2020).

2. Sun Z, et al. Chemopreventive and chemotherapeutic actions of mTOR inhibitor in genetically defined head and neck squamous cell carcinoma mouse model. Clin. Cancer Res. **18**, 5304-13 (2012).

3. Wang Z, et al. Syngeneic animal models of tobacco-associated oral cancer reveal the activity of in situ anti-CTLA-4. Nat. Commun. **10**, 5546 (2019).

4. Zheng DW, et al. Biomaterial-mediated modulation of oral microbiota synergizes with PD-1 blockade in mice with oral squamous cell carcinoma. Nat. Biomed. Eng. **6**, 32-43 (2022).

5. Wu L, et al. Blockade of TIGIT/CD155 Signaling Reverses T-cell Exhaustion and Enhances Antitumor Capability in Head and Neck Squamous Cell Carcinoma. Cancer Immunol. Res. **7**, 1700-13 (2019).

6. Lohavanichbutr P, et al. A 13-gene signature prognostic of HPV-negative OSCC: discovery and external validation. Clin. Cancer Res. **19**, 1197-203 (2013).

7. Tan J, et al. Association study of genetic variation in the autophagy lysosome pathway genes and risk of eight kinds of cancers. Int. J. Cancer. **143**, 80-7 (2018).

8. Yu G, Wang L, Han Y, He Q. clusterProfiler: an R package for comparing biological themes among gene clusters. OMICS. **16**, 284-7 (2012).

9. Wu T, et al. clusterProfiler 4.0: A universal enrichment tool for interpreting omics data. Innovation (Cambridge (Mass.)). **2**, 100141 (2021).

10. Shannon P, et al. Cytoscape: a software environment for integrated models of biomolecular interaction networks. Genome Res. **13**, 2498-504 (2003).

11. Smoot M, Ono K, Ruscheinski J, Wang P, Ideker T. Cytoscape 2.8: new features for data integration and network visualization. Bioinformatics. **27**, 431-2 (2011).

12. Therneau TM. A Package for Survival Analysis in S version 2.38. <https://CRAN.R-project.org/> (2015).

13. Mootha V, et al. PGC-1alpha-responsive genes involved in oxidative phosphorylation are coordinately downregulated in human diabetes. Nat. Genet. **34**, 267-73 (2003).

14. Subramanian A, et al. Gene set enrichment analysis: a knowledge-based approach for interpreting genome-wide expression profiles. Proc. Natl. Acad. Sci. U. S. A. **102**, 15545-50 (2005).

15. Barbie D, et al. Systematic RNA interference reveals that oncogenic KRAS-driven cancers require TBK1. Nature. **462**, 108-12 (2009).

16. Jiang P, et al. Signatures of T cell dysfunction and exclusion predict cancer immunotherapy response. Nat. Med. **24**, 1550-8 (2018).

17. Newman A, et al. Robust enumeration of cell subsets from tissue expression profiles. Nat. Methods. **12**, 453-7 (2015).

18. Li T, et al. TIMER2.0 for analysis of tumor-infiltrating immune cells. Nucleic Acids Res. **48**, W509-W14 (2020).

19. Li T, et al. TIMER: A Web Server for Comprehensive Analysis of Tumor-Infiltrating Immune Cells. Cancer Res. **77**, e108-e10 (2017).
