# Supplementary material for: LIMP-2 enhances cancer stem-like cell properties by promoting autophagy-induced GSK3β degradation in head and neck squamous cell carcinoma
Source: Int J Oral Sci. 2023 Jun 8;15:24. doi: 10.1038/s41368-023-00229-0 (PMC10250453; doi:10.1038/s41368-023-00229-0)
Supplement: Supplementary file 2 — Supplementary Figures [file 41368_2023_229_MOESM2_ESM.docx]

**LIMP-2 enhances cancer stem-like cell properties by promoting autophagy-induced GSK3β degradation in head and neck squamous cell carcinoma**

Yuan-Tong Liu^1^, Shu-Jin Li^1^, Shuo Wang^1^, Qi-Chao Yang^1^, Zhi-Zhong Wu^1^, Meng-Jie Zhang^1^, Lei Chen^1^, Zhi-Jun Sun^1, 2, *^

^1^ The State Key Laboratory Breeding Base of Basic Science of Stomatology (Hubei-MOST) & Key Laboratory for Oral Biomedicine Ministry of Education, School and Hospital of Stomatology, Wuhan University, Wuhan, China.

^2^ Department of Oral Maxillofacial-Head Neck Oncology, School and Hospital of Stomatology, Wuhan University, Wuhan, China.

*Corresponding author:

Zhijun Sun, School and Hospital of Stomatology, Wuhan University, Wuhan 430079, China; Email: sunzj@whu.edu.cn.

The authors declare no potential conflicts of interest.

**Supplementary Figures and Figure Legends**


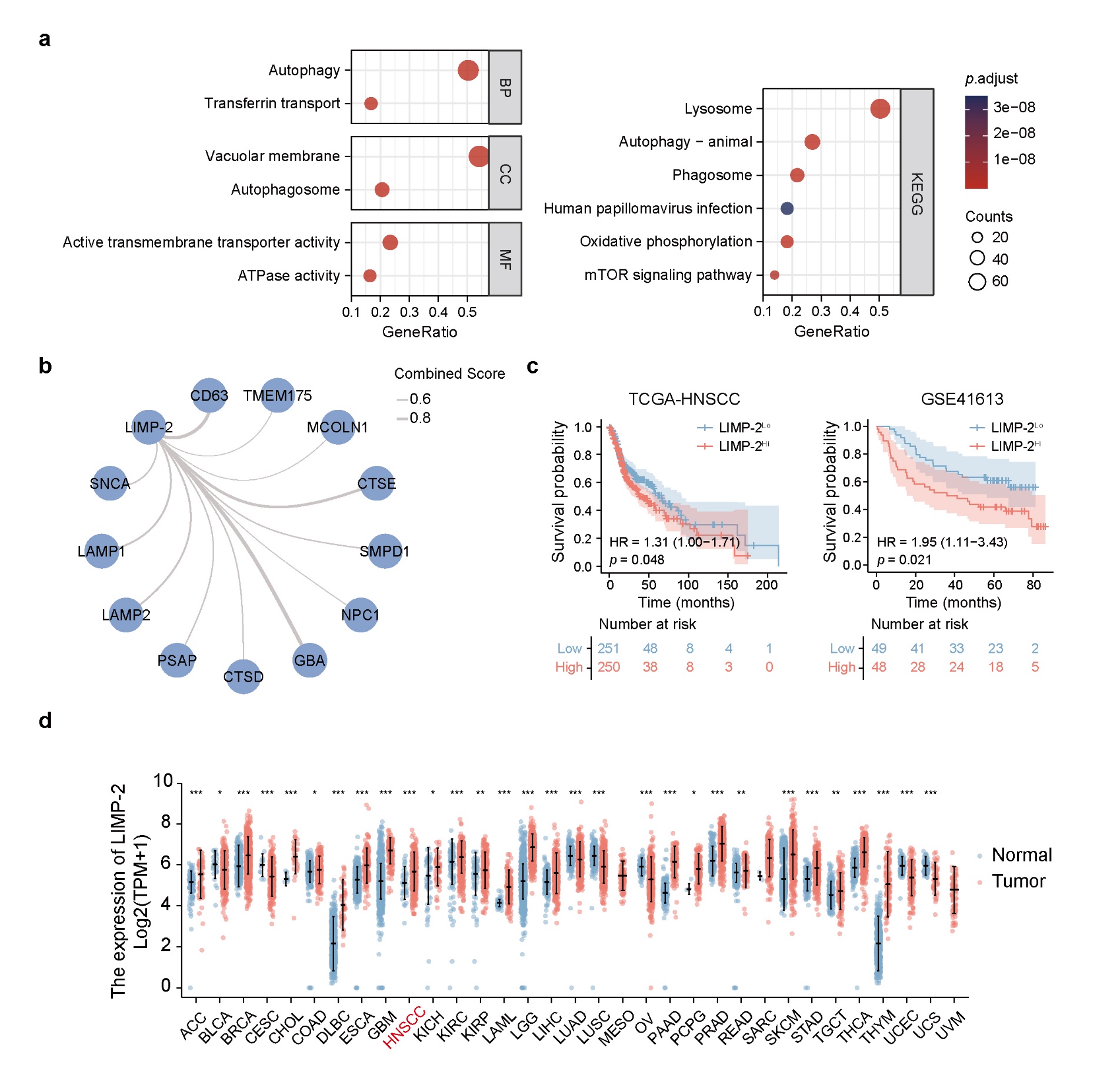
**Supplementary Fig. 1.** **a** Gene Ontology (GO) and Kyoto Encyclopedia of Genes and Genomes (KEGG) analyses of the autophagy‒lysosome gene. **b** The protein‒protein interaction network of LIMP-2 and genes related to the autophagy‒lysosome pathway was visualized by using the STRING database. **c** Kaplan‒Meier survival analysis suggests that high expression of LIMP-2 was associated with poor prognosis at the median cutoff value in the TCGA-HNSCC dataset and GSE41613 dataset. **d** The analysis of the TCGA and GTEx datasets indicated that LIMP-2 expression was significantly upregulated in 23 cancers, including HNSCC. ^*^*p* < 0.05, ^**^*p* < 0.01, ^***^*p* < 0.001.


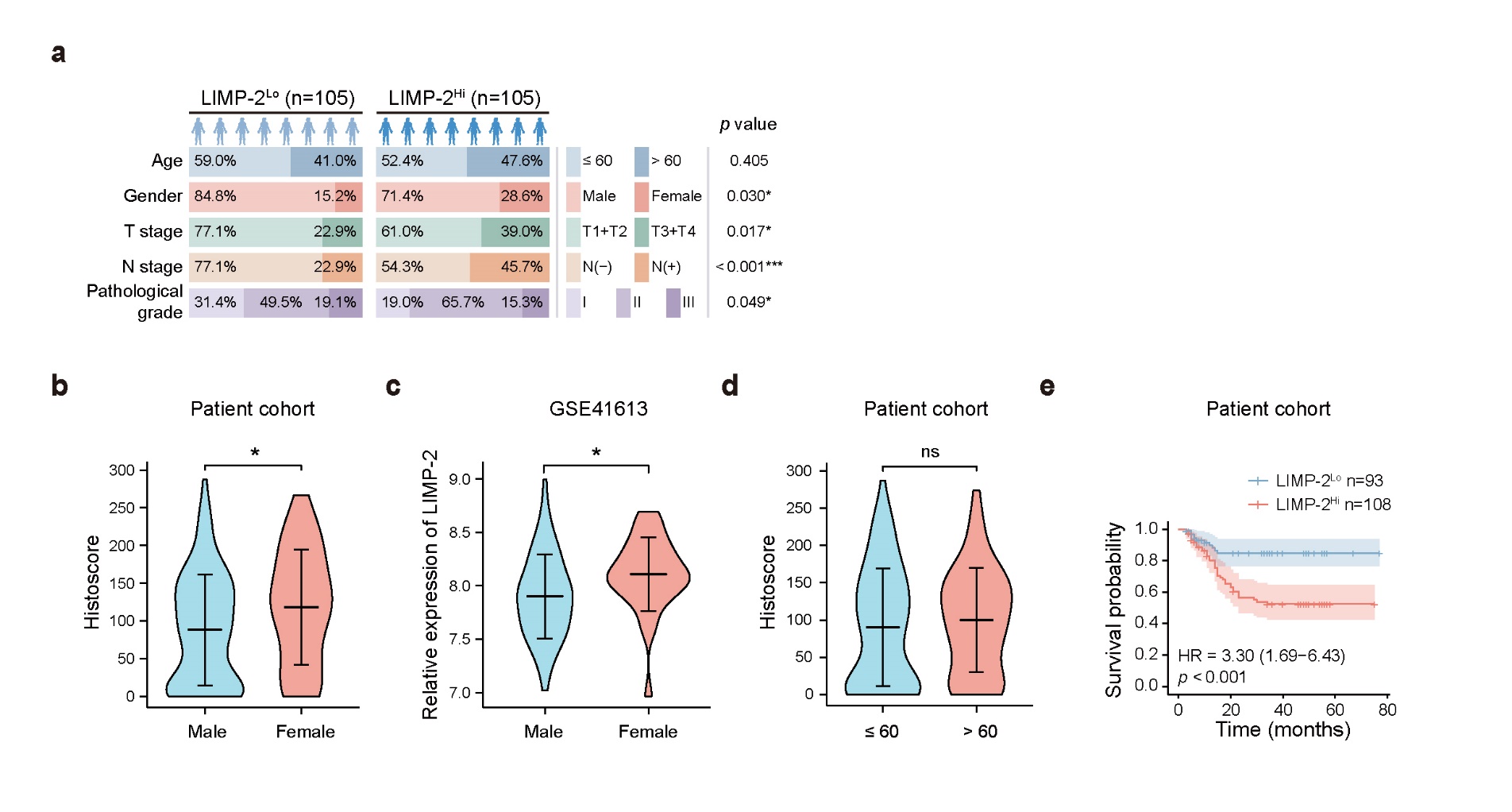


**Supplementary Fig. 2.** **a** Proportional differences in clinicopathological factors (including age, gender, T stage, N stage, and pathological grade) in the LIMP-2 ^high^ and LIMP-2 ^low^ expression groups from our patient cohort. The χ^2^ test was performed to assess the relationship between LIMP-2 and clinical characteristics. **b** Quantification of LIMP-2 expression between males (n=164) and females (n=46) in our patient cohort. **c** Quantification of LIMP-2 expression between males (n=66) and females (n=31) in the GSE41613 dataset. **d** Quantification of LIMP-2 expression between age ≤ 60 (n=117) and age＞60 (n=93) in our patient cohort. **e** Kaplan‒Meier survival analysis suggests that high expression of LIMP-2 was associated with poor prognosis at the best cutoff value in our patient cohort. ^*^*p*< 0.05, ^**^*p* < 0.01, ^***^*p* < 0.001, and ns represents no significance.


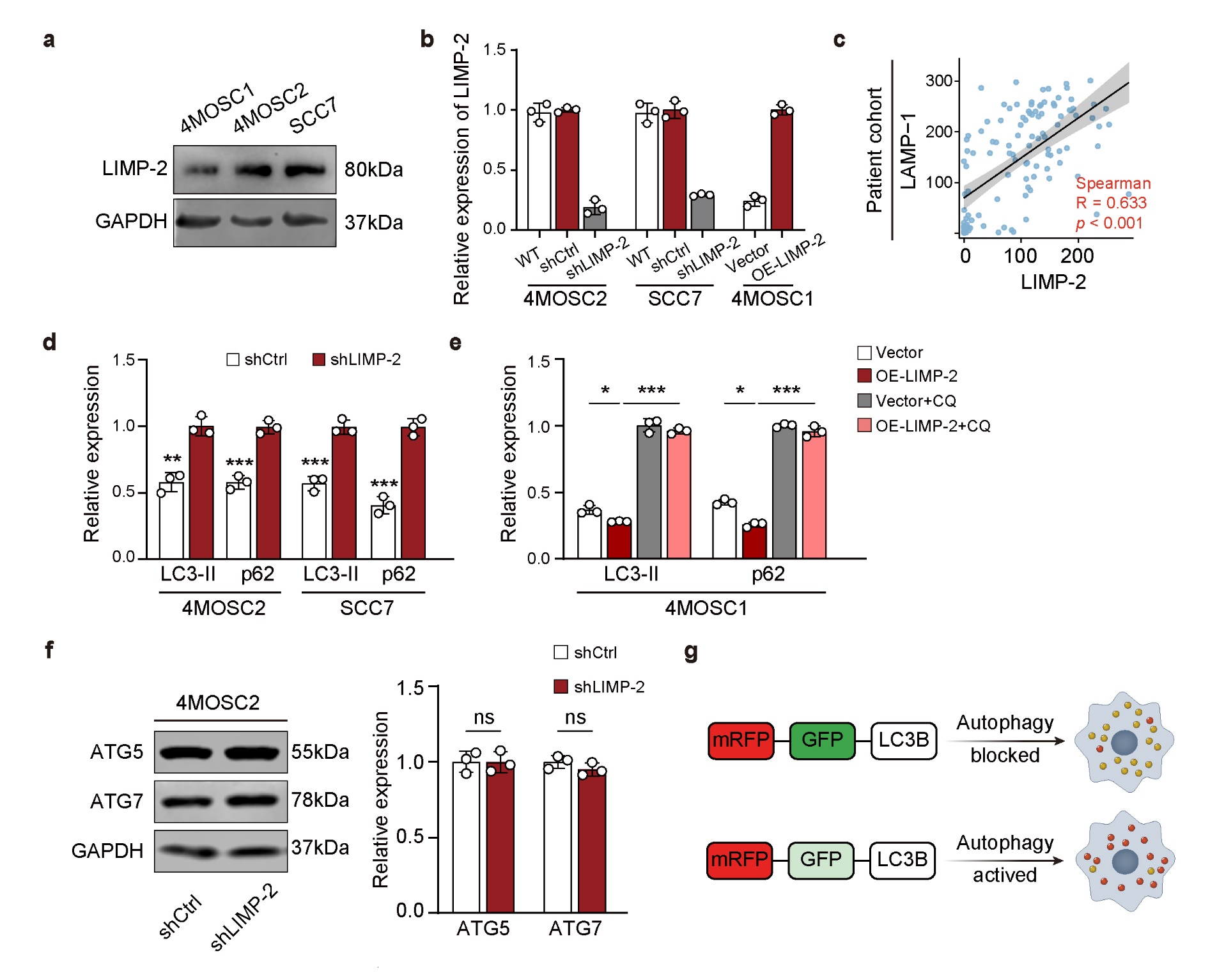
**Supplementary Fig. 3.** **a** Western blot analysis of LIMP-2 expression in mice HNSCC cell lines (4MOSC1, 4MOSC2, and SCC7). **b** Overexpression of LIMP-2 in 4MOSC1 cells, and knockdown of LIMP-2 by shLIMP-2 in 4MOSC2 and SCC7 cells were confirmed by western blot analysis. **c** Spearman's correlation between LIMP-2 expression with LAMP-1 in our patient cohort. **d** Western blot analysis showed LIMP-2 knockdown enhanced expression of LC3-II and p62 compared to those in the negative control groups in 4MOSC2 and SCC7 cells. **e** Western blot analysis of LC3-II, p62, and LIMP-2 in vector and OE-LIMP-2 4MOSC1 cells with or without the autophagy inhibitor chloroquine (CQ). **f** The expression of ATG5 and ATG7 in 4MOSC2 of different groups (shCtrl, shLIMP-2) were analyzed by western blot. **g** The mRFP-GFP-LC3 plasmid was transfected to detect autophagic flux. All results were calculated in at least three independent experiments and expressed as mean ± SD. ^*^*p*< 0.05, ^**^*p* < 0.01, ^***^*p* < 0.001, and ns represents no significance.


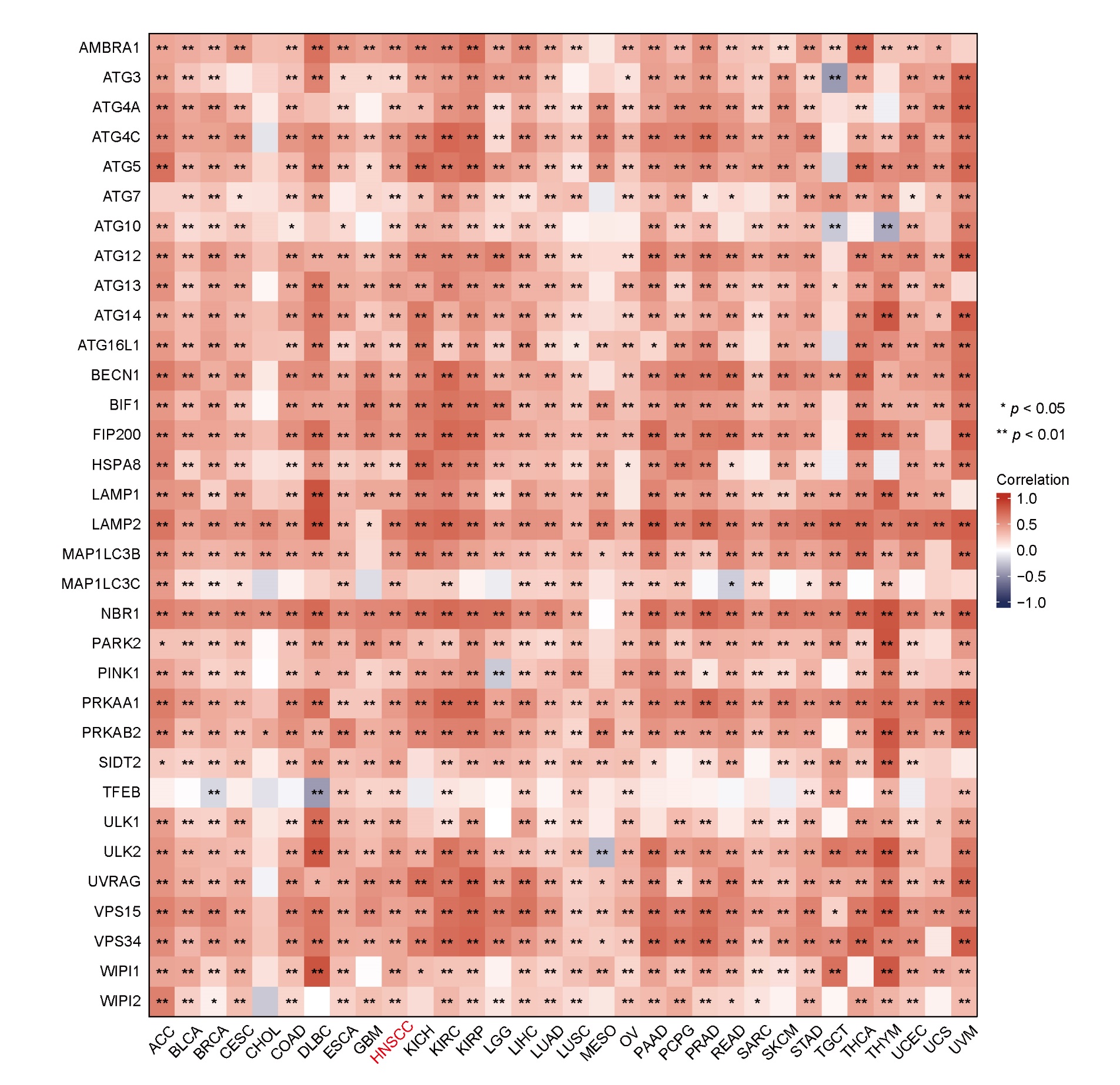
**Supplementary Fig. 4.** Spearman’s correlation between LIMP-2 and autophagy-related genes across cancers.


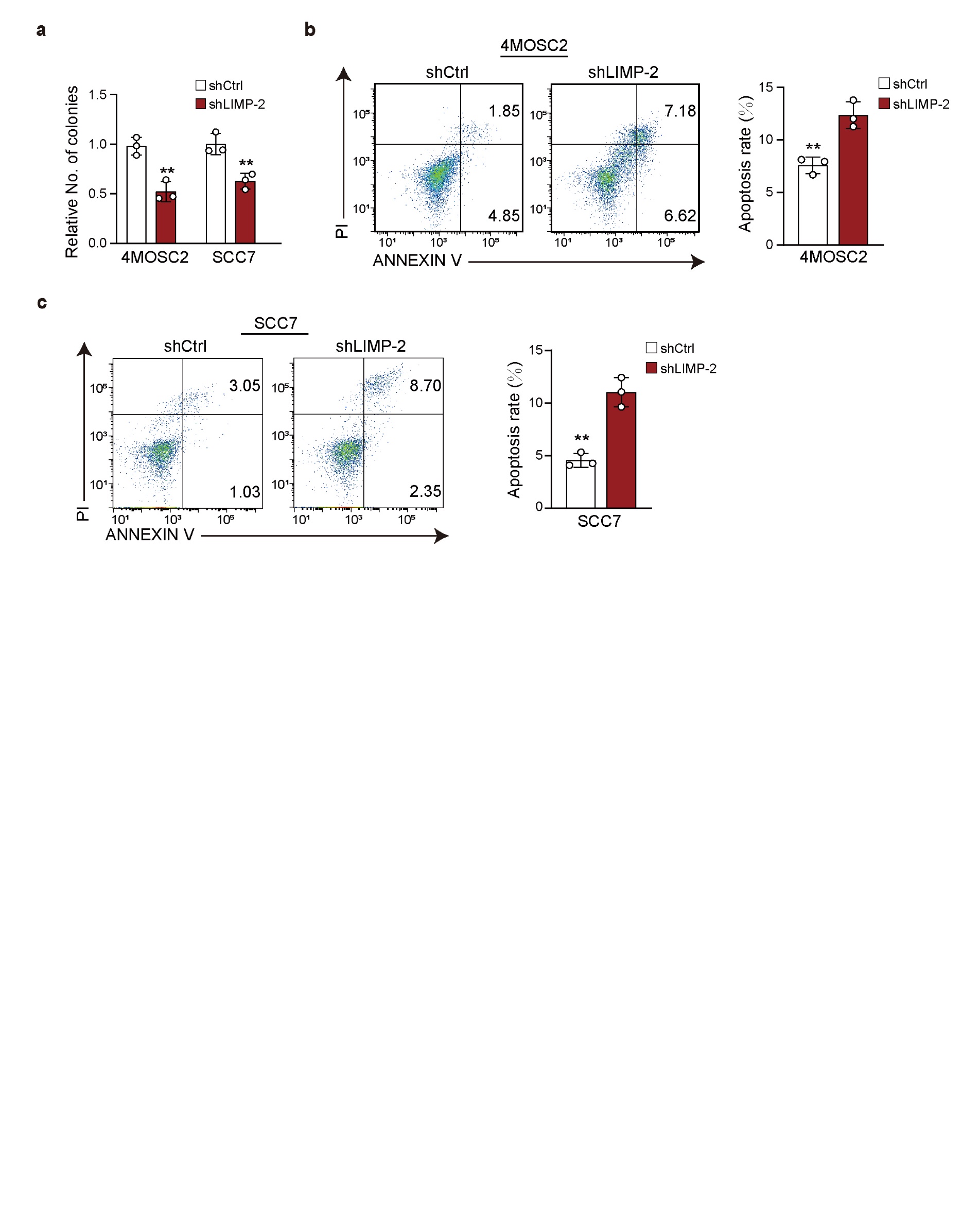
**Supplementary Fig. 5. a** Quantification of stained colonies in shLIMP-2 and shCtrl cells. **b, c** Quantification of apoptotic ratio according to flow cytometry analysis of (**b**) 4MOSC2 and (**c**) SCC7 cells upon LIMP-2 knockdown. All results were calculated in at least three independent experiments and expressed as mean ± SD. ^*^*p* < 0.05, ^**^*p* < 0.01, ^***^*p* < 0.001.


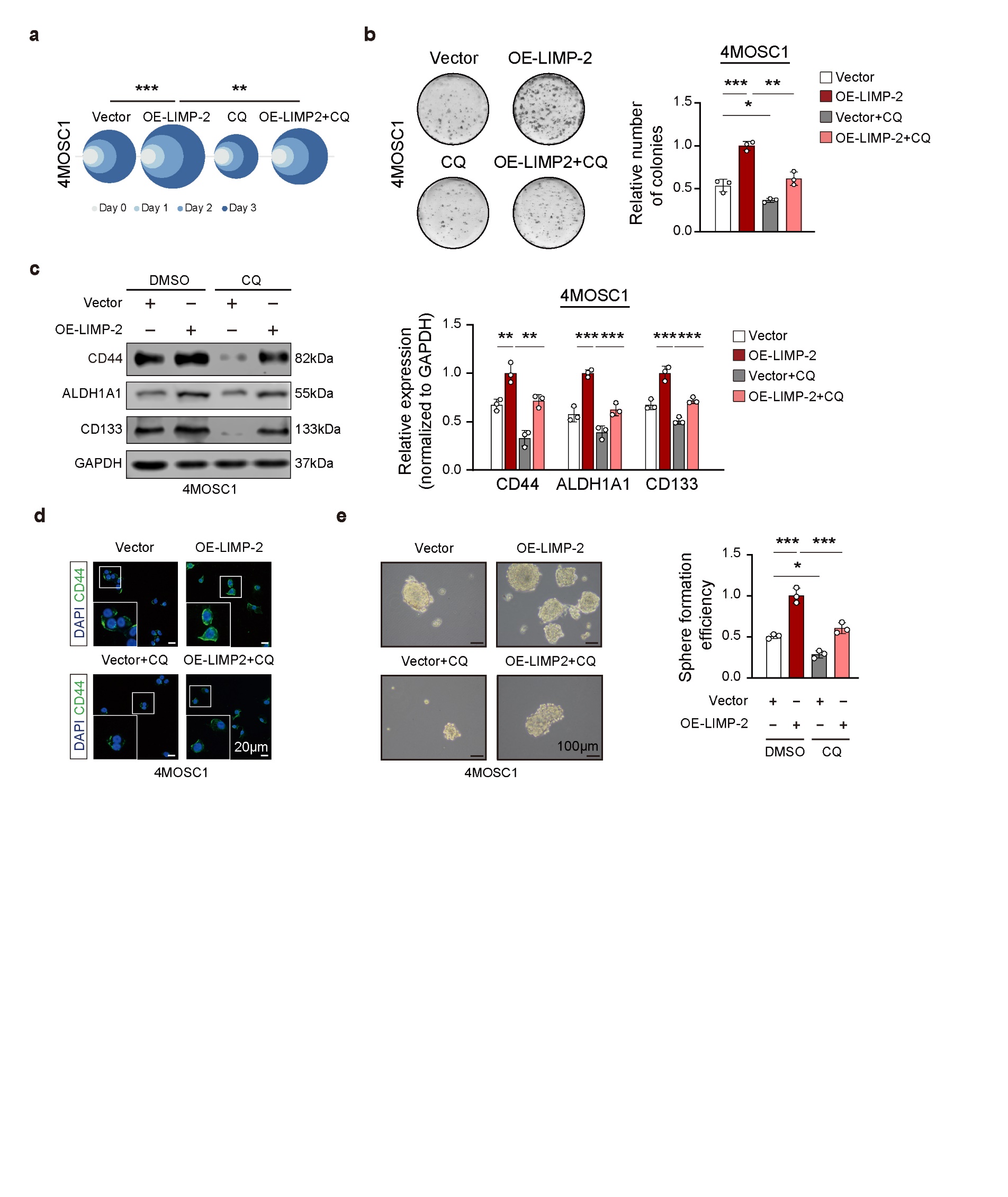
**Supplementary Fig. 6.** **a, b** The proliferation of vector and OE-LIMP-2 4MOSC1 cells treated with DMSO or CQ was examined by CCK8 assay (**a**) and colony formation assay (**b**). **c** Western blot results showed that LIMP-2 overexpression increased the expression of CSC markers (CD44, ALDH1A1, CD133) in 4MOSC1 cells. Additionally, CQ partially inhibited the effect of LIMP-2 on HNSCC stemness *in vitro*. **d** Representative immunofluorescence staining of CD44. **e** The stemness of 4MOSC1 cells was examined by tumor sphere formation assay. All results were calculated in at least three independent experiments and expressed as mean ± SD. ^*^*p* < 0.05, ^**^*p* < 0.01, ^***^*p* < 0.001.


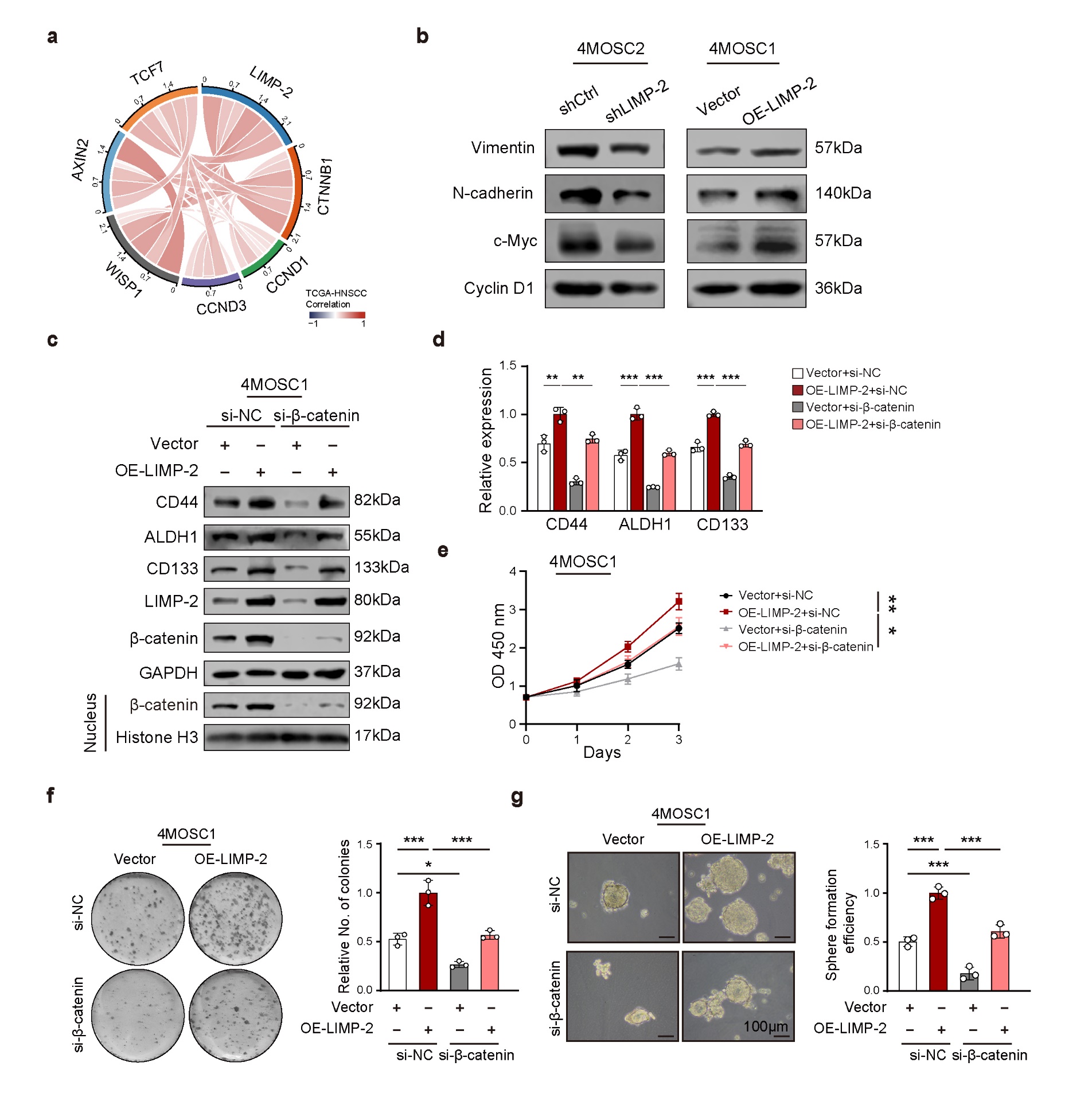
**Supplementary Fig. 7.** **a** Spearman's correlation between LIMP-2 expression with Wnt pathway-related genes in TCGA-HNSCC dataset. **b** Western blot results and quantification of Wnt pathway-related genes in indicated cell lines. **c, d** Western blot assays indicated that β-catenin knockdown abolished the promoting effects of LIMP-2 on the expression of CSC-related markers. Histone H3 was loaded as a nuclear marker. **e, f** The proliferation of vector and OE-LIMP-2 4MOSC1 cells treated with siNC or siβ-catenin was examined by CCK-8 assay (**e**) and colony formation assay (**f**). **g** The stemness of 4MOSC1 cells was examined by tumor sphere formation assay. All results were calculated in at least three independent experiments and expressed as mean ± SD. ^*^*p* < 0.05, ^**^*p* < 0.01, ^***^*p* < 0.001.


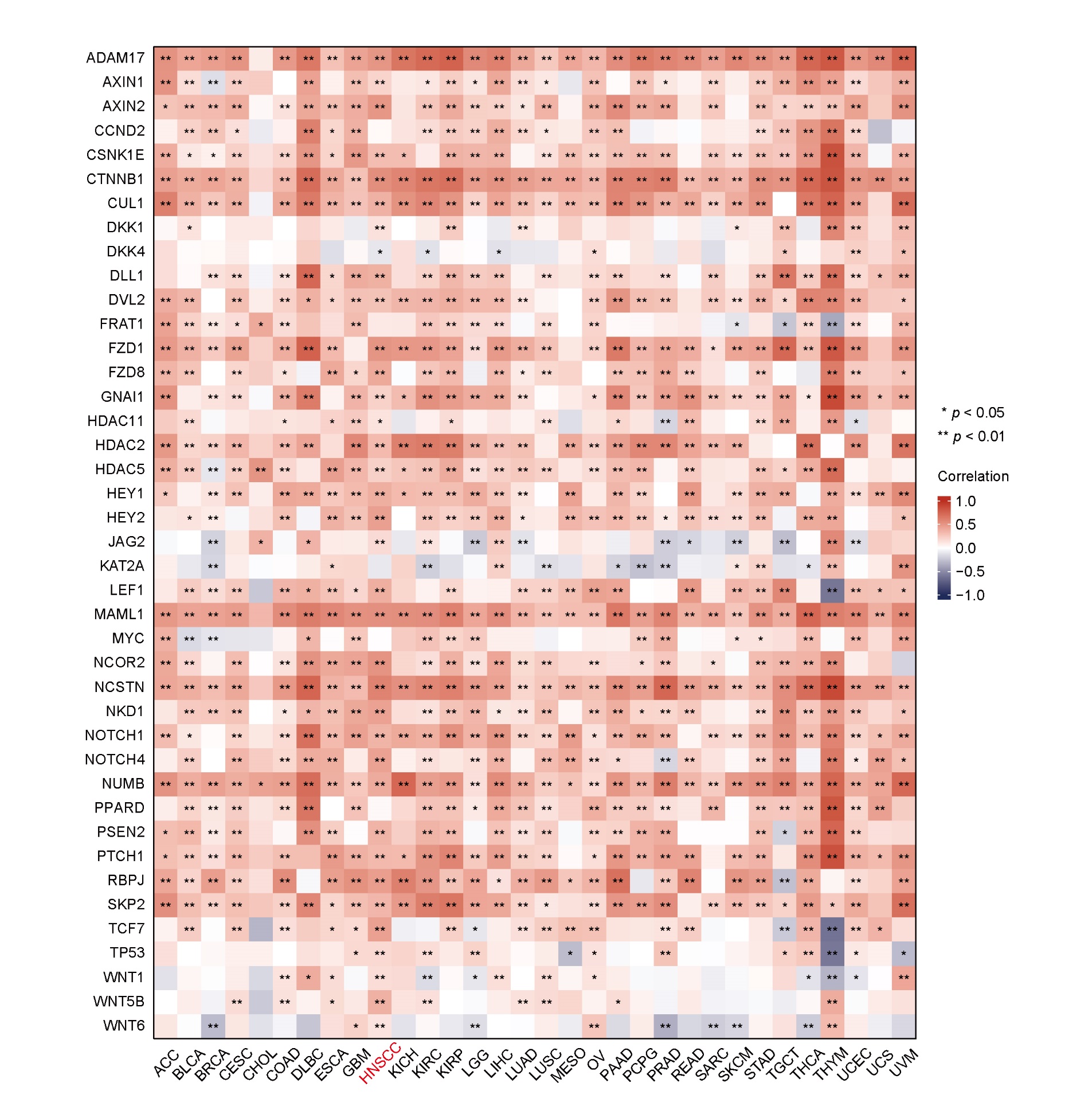
**Supplementary Fig. 8.** Spearman’s correlation between LIMP-2 and Wnt pathway-related genes across cancers.


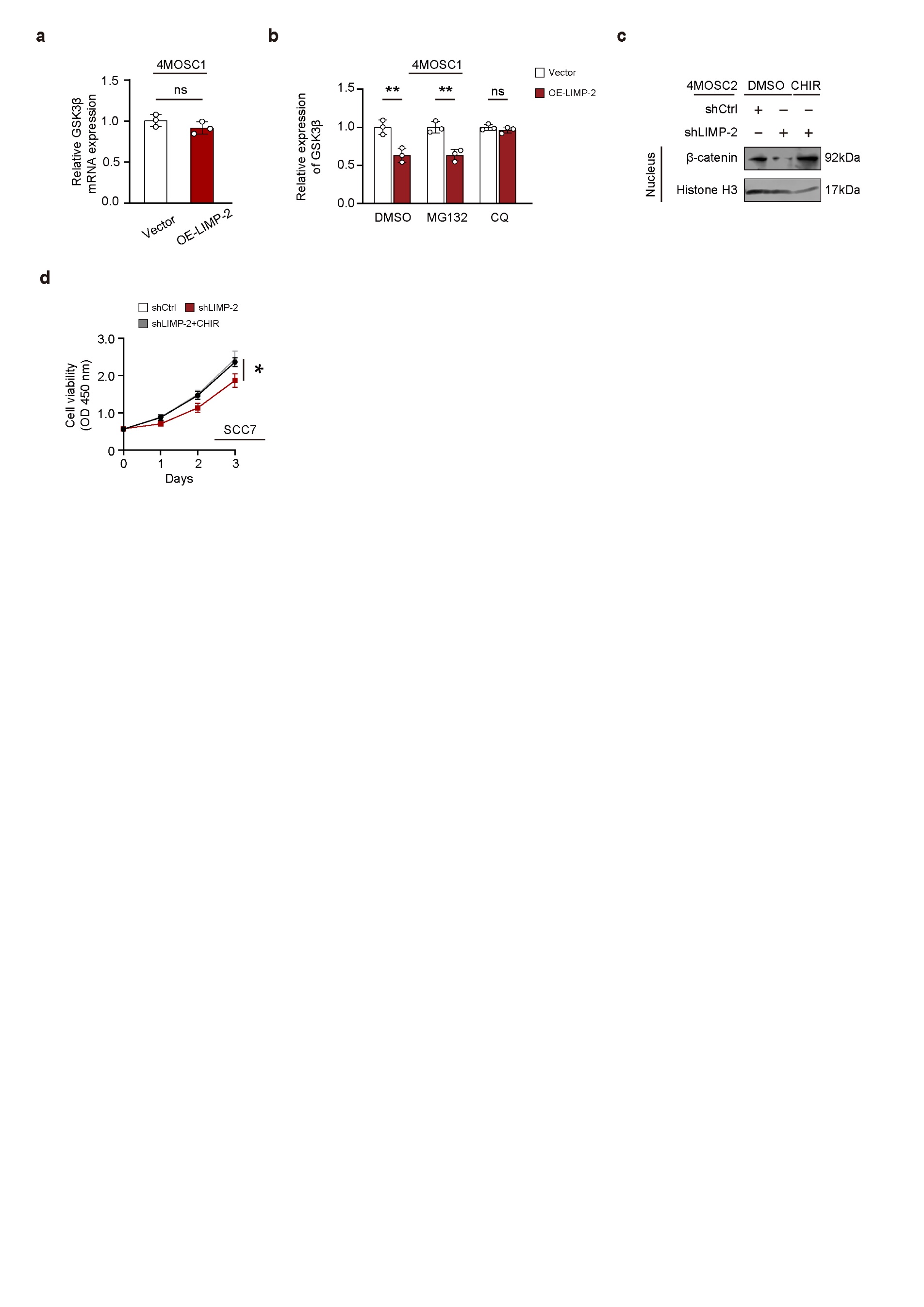
**Supplementary Fig. 9.** **a** RT-qPCR analysis of GSK3β level in 4MOSC1 cells transfected with pcDNA3.1/LIMP-2 versus pcDNA3.1 vector. **b** Quantification of GSK3β in control (empty vector) or LIMP-2-overexpressing 4MOSC1 cells treated with DMSO, MG132, or CQ. **c** Western blot results of shLIMP-2 4MOSC2 cells treated with specific inhibitor of GSK3β (CHIR). **d** Proliferation of shLIMP-2 SCC7 cells treated with CHIR was examined by CCK-8 assay. All results were calculated in at least three independent experiments and expressed as mean ± SD. ^*^*p* < 0.05, ^**^*p* < 0.01, ^***^*p* < 0.001, and ns represents no significance.


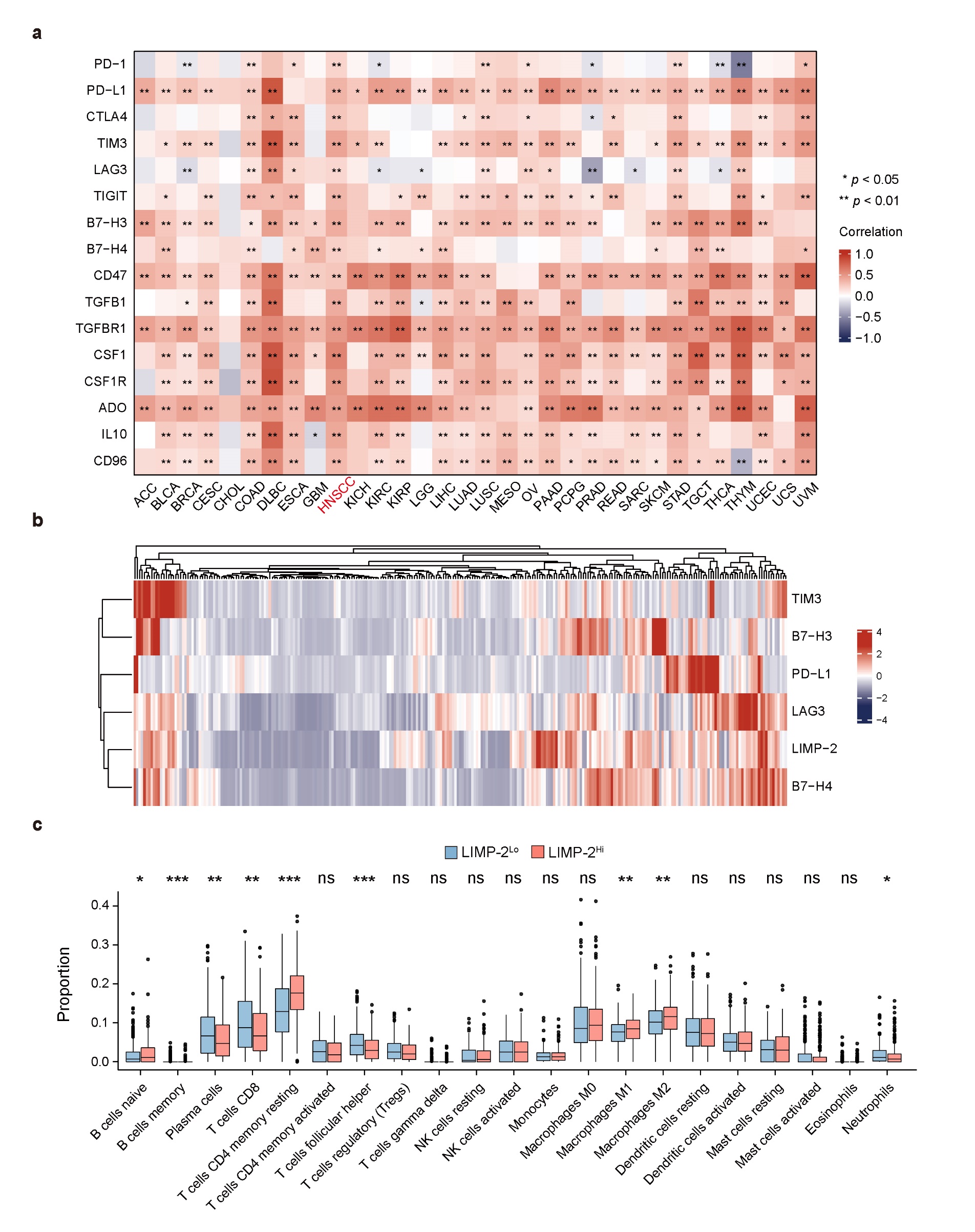
**Supplementary Fig. 10.** **a** Spearman’s correlation between LIMP-2 and immune checkpoints in pan-cancer. **b** Hierarchical clustering shows the affinitive protein expression correlation of LIMP-2, PD-L1, TIM3, LAG3, B7-H3, and B7-H4 in human HNSCC. **c** The proportions of TME cells in different LIMP-2 subgroups. Significant statistical differences between the two subgroups were evaluated using the Wilcoxon test. ^*^*p* < 0.05, ^**^*p* < 0.01, ^***^*p* < 0.001, and ns represents no significance.


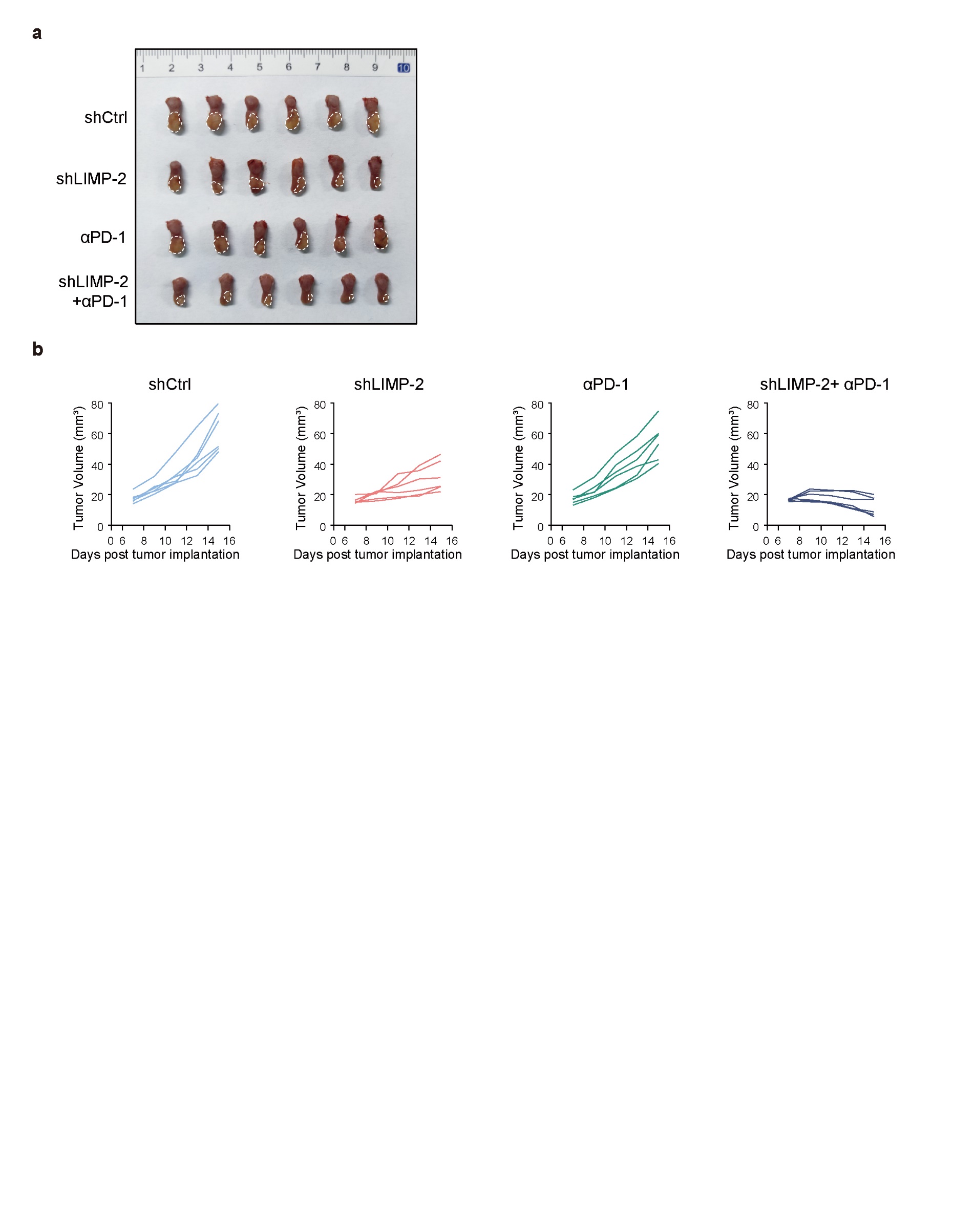
**Supplementary Fig. 11.** **a, b** Tumor image and tumor growth curves of different treatment groups.
